# *AGAMOUS* Gene as a New Sex-Identification Marker in Fig (*Ficus carica* L.) Is More Efficient Than *RAN1*

**DOI:** 10.3389/fpls.2021.755358

**Published:** 2021-10-20

**Authors:** Xu Wang, Miaoyu Song, Moshe A. Flaishman, Shangwu Chen, Huiqin Ma

**Affiliations:** ^1^Department of Fruit Tree Sciences, College of Horticulture, China Agricultural University, Beijing, China; ^2^Department of Fruit Tree Sciences, Agricultural Research Organization, The Volcani Center, Bet Dagan, Israel; ^3^College of Food Science and Nutrition Engineering, China Agricultural University, Beijing, China

**Keywords:** *FcAG*, *FcRAN1*, molecular marker, sex identification, *Ficus carica* L.

## Abstract

Fig is an ancient gynodioecious fruit tree with females for commercial fruit production and hermaphrodites (males) sometimes used as pollen providers. An early sex-identification method would improve breeding efficiency. Three *AGAMOUS* (*AG*) genes were recruited from the *Ficus carica* genome using *AG* sequences from *Ficus microcarpa* and *Ficus hispida*. *FcAG* was 5230 bp in length, with 7 exons and 6 introns, and a 744-bp coding sequence. The gene was present in both female and male fig genomes, with a 15-bp deletion in the 7th exon. The other two AG genes (*FcAG2-Gall_Stamen* and *FcAG3-Gall_Stamen*) were male-specific, without the 15-bp deletion (759-bp coding sequence), and were only expressed in the gall and stamen of the male fig fruit. Using the deletion as the forward primer (AG-Marker), male plants were very efficiently identified by the presence of a 146-bp PCR product. The previously reported fig male and female polymorphism gene *RESPONSIVE-TO-ANTAGONIST1* (*RAN1*) was also cloned and compared between male and female plants. Fifteen SNPs were found in the 3015-bp protein-coding sequence. Among them, 12 SNPs were identified as having sex-differentiating capacity by checking the sequences of 27 known male and 24 known female cultivars. A RAN1-Marker of 608 bp, including 6 SNPs, was designed, and a PCR and sequencing-based method was verified with 352 fig seedlings from two hybrid populations. Our results confirmed that the newly established AG-Marker is as accurate as the RAN1-Marker, and provide new clues to understanding *Ficus* sex determination.

## Introduction

Fig (*Ficus carica* L., Moraceae) is regarded as one of the earliest domesticated fruit trees, widely cultivated in the Mediterranean region, and in other parts of the world (Kislev et al., 2006). As with other species in the genus *Ficus*, the fig is gynodioecious. The trees can be divided into female figs and caprifigs (usually called male figs) (Ikegami et al., 2013). The female figs bear edible fruit. Upon ripening, the long-styled female flowers (pistils) develop inside the syconium, making up most of the edible part of the fruit (Stover et al., 2007). The caprifig is not usually edible, but stamens growing inside the syconia produce pollen, and the quality and storability of female fig fruit improve after pollination (Lama et al., 2020; Marcotuli et al., 2020). In addition, there are short-styled female flowers (galls) in the syconia of caprifigs that can serve as hosts for fig-wasp (*Blastophaga psenes* L.) larvae (Gu and Yang, 2013; Zhang et al., 2020; Wang et al., 2021).

The evolution of fig from monoecious to dioecious was the first step in the double mutation model (Charlesworth and Charlesworth, 1978; Charlesworth, 2016). A recessive homozygous mutation appeared in wild fig trees (caprifigs), which resulted in male flower abortion but retention of female function, resulting in fig trees with all female flowers and significantly improved fruit taste. To date, no dominant mutation has been found that suppresses female function, resulting in all male individuals.

Key genes regulating plant sex differentiation are of high interest in the plant sciences. Examples of these include: the candidate male-determining gene *OGI* and the anther fertility-regulating gene *MeGI* found in persimmon (*Diospyros lotus*) (Akagi et al., 2014); the regulators of male flower development *CYP703* and *GPAT3* identified in *Phoenix dactylifera* (Torres et al., 2018); the feminization-suppressing gene *SyGI* and male-function-maintaining gene *FrBy* reported in kiwifruit (Akagi et al., 2019); a mutation of gene *INP1* in *Vitis vinifera* resulting in male sterility of grape; the transcription factor *YABBY3* in *V. vinifera* found to be associated with female sterility (Massonnet et al., 2020); and the female-suppressor gene *SOFF* and the male-promoting gene *aspTDF1* identified in *Asparagus officinalis* (Harkess et al., 2020).

According to previous reports, the short-styled female flower (gall, G) is dominant over long-styled female flower (pistil, g), and stamen presence (A) is dominant over stamen absence (a) (Storey, 1975; Stover et al., 2007). Crossing between caprifigs can produce fertile offspring—a prerequisite for the shift from monoecy to the development of all female figs. A caprifig with a recessive mutation (GA/ga) will produce 25% recessive homozygous offspring, i.e., female figs (ga/ga) (Mori et al., 2017). The GA and ga alleles always appear in pairs, and there is a linkage in the key genes regulating floral organ differentiation.

The first-draft whole-genome sequence of fig *F. carica* was published in 2017 using female cv. Horaishi (Mori et al., 2017). Restriction-site-associated sequencing technique and genome-wide association analysis were used to identify the candidate sex-determining gene *RESPONSIVE-TO-ANTAGONIST1* (*RAN1*), located on the seq000259 scaffold in the 9.2–12.1 cM region of the Fc01a linkage group. Two missense mutations in *RAN1* were found to be strongly associated with fig sex phenotypes. The *FcRAN1* gene had 9 exons, and a 3015-bp protein-coding sequence. *FcRAN1* was the homolog of a copper-transporting ATPase, and the gene was also named *Heavy Metal ATPase 7* (*HMA7*). In *Arabidopsis thaliana*, *RAN1* was located on the Golgi membrane, and was responsible for transporting copper ions to ethylene receptors and activating them (Hirayama et al., 1999; Woeste and Kieber, 2000; Binder et al., 2010). *FcRAN1* was preliminarily speculated to be the sex-determining *A* gene for figs, and a cleaved amplified polymorphism sequence (CAPS)-based method was established (Mori et al., 2017). Ikten and Yilmaz (2019) verified the CAPS method and found that restriction endonuclease *Pci*I provides 100% sex discrimination for PCR products, while restriction endonuclease *Hpy*CH4IV gave some false-positive results. Nevertheless, restriction endonuclease digestion is time-consuming, the required enzymes are expensive, and well-controlled conditions are needed for successful performance.

The role of *RAN1* in fig sex determination was challenged by Zhang et al. (2020). Whole-genome sequencing and assembly of two *Ficus* species—*F. microcarpa* (monoecious) and *F. hispida* (functional dioecious)—revealed a male-specific sex-determination candidate gene *FhAG2* (floral homeotic protein AGAMOUS) (Zhang et al., 2020). *AtAG* belongs to the C-class of the MADS-box gene family, which has been confirmed to regulate the determination of stamen, carpel and floral meristem (Dreni and Kater, 2014). Transcriptome data showed that *FhAG2* was only highly expressed in the stamens of male *F. hispida* syconia; *FhAG3*, which was not anchored to any chromosome, was also specifically expressed in stamens. The results strongly suggested that *FhAG2* is the critical sex-determination gene (Zhang et al., 2020).

In this study, we tried to establish a convenient, reliable and rapid molecular marker for the identification of male and female figs, without relying on rare and expensive restriction endonucleases. Both *AG* and *RAN1* were cloned and their sequences were compared using known-sex cultivars and hybrid progeny, resulting in the development of two markers. Our results confirmed *FcAG* as a new sex-identification marker, and the possible involvement of *AG* and *RAN1* in fig sex determination is discussed.

## Materials and Methods

### Plant Materials and Preparation of Amplification Templates

In this study, leaf, root, stem, and fruit were collected from 27 male and 24 female fig cultivars. Leaf and fruit of two F1 hybrid populations (group 207, *n* = 263; group 205, *n* = 89) were also used. The detailed list of materials is shown in Supplementary Table 1. Pistil, stamen, gall, and peel were separated from female and male fig fruit, and all collected fresh materials were quickly frozen in liquid nitrogen, fully ground, and stored at −80°C. The genomic DNA (gDNA) and total RNA were extracted by the CTAB method as described previously (Cui et al., 2021); concentration and purity were measured in a NanoDrop2000 spectrophotometer (Thermo Scientific, United States). Integrity of gDNA and total RNA was checked by 1% agarose gel (Biowest, Spain) electrophoresis (Reid et al., 2006; Chai et al., 2019), and RNA concentration was normalized (Wang et al., 2019). Then the total RNA was treated with DNase I (D2270A, Takara, Japan) at 37°C for 30 min to remove any contaminating DNA, and mRNA was then isolated from 2 μg total RNA using cellulose oligo (dT) magnetic beads (3806, Takara, Japan); cDNA was synthesized with a cDNA Synthesis Kit (639552, Takara, Japan).

### Primer Sequence Design

Specific primers AG-F and AG-R were designed according to the coding sequences (CDSs) of the genus *Ficus* (*F. microcarpa*, *F. hispida*, and *F. carica*) (Table 1). AG-Marker was a pair of male-specific primers for *FcAG2-Gall_Stamen* and *FcAG3-Gall_Stamen*. FcRAN1 was the full-length gene primer pair based on the *FcRAN1* cDNA sequences of female fig “Horaishi” and “Caprifig6085” reported by Mori et al. (2017), and the quantitative real-time PCR (qRT-PCR) primers of *FcRAN1* were designed from the non-mutated region of the 7th exon. The single nucleotide polymorphism (SNP)-identification primers 6SNP-1 (RAN1-Marker), 1-SNP, 6SNP-2, and 2-SNP were designed with 6, 1, 6, and 2 SNP sites in their amplified fragments, respectively. Actin was used as the housekeeping gene following Freiman et al. (2015).

**TABLE 1:** Primers used in this study.

Primer name	Primer sequence (5′→3′)	Length (bp)	Target length (bp)
AG-F	ATGKCGTWCCAAAACAAGGWGAWGA	25	759
AG-R	TTASACTAATTGGAGGGCCATGGRA	25	
AG-Marker-F	CAGGAGGAGGAAGCT	15	146
AG-Marker-R	TTACACTAATTGGAGGGCCA	20	
FcRAN1-F	ATGGCGGCGAGCGTCCGACA	20	3015
FcRAN1-R	CTATTCTACAGTTATTTCTAGTATA	25	
RAN1-Marker-F	ATATCAAGAATGCAATCGAGGA	22	608
RAN1-Marker-R	GCTGAGAAATAGACTAGAGATG	22	
qPCR-F	CCACTGGCAAAGGCAATAGT	20	156
qPCR-R	TTGAACTCCTCTGCCGGGAA	20	
1-SNP-F	TCCTGTCTTTCTCATACGAGTAGTT	25	371
1-SNP-R	TGGCGTCAGATGTTTTCCCC	20	
6SNP-2-F	CTGGAGCTCTTCGTGCTTAC	20	766
6SNP-2-R	AAGCATTGAACTCCTCTGCCG	21	
2-SNP-F	TCACGATGTAAGGGCAGAGG	20	444
2-SNP-R	GTGGTCAGCCTTGGTTTTCTG	21	
Actin-F	GAACCACCAGACAGGACGATG	21	250
Actin-R	CTACCACTGCTGAACGGGAAA	21	

*AG primers contain degenerate bases: K(G/T), W(A/T), S(G/C), R(A/G).*

### PCR and Quantitative Real Time-PCR

High-fidelity enzyme (KOD-Plus-Neo, TOYOBO, Japan) was used for all PCRs related to gene cloning, sequencing and SNP-site identification. PCR amplification was carried out in a Model T100 thermal cycler (BioRad, United States). The PCR products were first checked by electrophoresis, then collected with an agarose gel purification and extraction kit (AP-GX-50, Axygen, United States). The fragments were individually ligated into the pClone007 vector (pClone007 Blunt Simple Vector Kit; Tsingke, China), and transformed into *Escherichia coli* DH5α cells; at least 15 single clones were picked and at least 10 clones were plasmid-sequenced, for each replication. Three biological replicates were used if not otherwise indicated. T5 Super PCR Mix (Tsingke) was used for amplification without further sequencing. The PCR products were checked by 1% agarose gel electrophoresis. ChamQ Universal SYBR qPCR Master Mix (Q711, Vazyme, China) was used for the qRT-PCRs carried out with QuantStudio^TM^ 6 (ABI, United States). Relative expression was calculated by the 2^–ΔΔCt^ method (Chai et al., 2018, 2019).

### Bioinformatics Tools and Data Analysis

TBtools (Chen et al., 2020) was used for the following bioinformatics analyses: local sequence alignment (BLAST-BLAST GUI Wrapper – Several Sequences to a Big File), visualization of gene structure (Graphics – Bio Sequence Structure Illustrator – Gene Structure View), location of genes on chromosomes (Graphics – Show Genes on Chromosome – Gene Location Visualize), and chromosome collinearity analysis (Graphics – Synteny Visualization – One Step MCScanX and Dual Systeny Plot and Multiple Synteny Plot).

NCBI BLAST^1^ was used for DNA sequence alignment, and UniProt BLAST^2^ for protein sequence alignment. NCBI ORF Finder^3^ was used to find possible protein-coding sequences. Novopro^4^ was used for CDS translation. The phylogenetic tree was constructed using Mega software (Mega v. 11^5^) with maximum likelihood (ML) method, the Jones–Taylor–Thornton (JTT) model and 1000 bootstrap replications. DNAMAN software^6^ was used for sequence alignment. SnapGene software^7^ was used for SNP localization and sequence alignment.

The transcriptome data of fig cv. Purple Peel fruit at different developmental stages, previously produced by our laboratory, were used for data mining (SRA accession: PRJNA723733, NCBI) (Wang et al., 2017; Zhai et al., 2021). Accession numbers for *F. carica* cv. Horaishi and cv. Dottato genomes are GCA_002002945.1 and GCA_009761775.1, respectively, in NCBI GenBank (Mori et al., 2017; Usai et al., 2020); for *F. microcarpa* and *F. hispida* genomes: GWHABKV00000000.1 and GWHALOG00000000, respectively, in CNCB^8^ (Zhang et al., 2020); and for the *Morus alba* genome: GCA_012066045.3 in NCBI GenBank (Jiao et al., 2020). The published female fig cv. Dottato genome was used to predict *FcAG, FcAG2-Gall_Stamen* and *FcAG3-Gall_Stamen* gene structure. All nucleotide and protein sequences used in this study are provided in Supplementary Table 2.

## Results

### *FcAG* Identification and Sequence Alignment

Fifty-three AG-related proteins were predicted using Usai et al.’s (2020) published “Dottato” fig genome sequence, including one AG protein (Gene ID: *FCD_00034093*, Supplementary Table 3), one AG isoform X1 and 51 AGAMOUS-LIKE (AGL) proteins. Thirty-one AG-related genes were annotated from fruit transcriptome data of fig cv. Purple Peel, including one AG (Gene ID: *c23673_g1*, 991 bp, 744 bp ORF) and 30 AGLs (Supplementary Table 3). These proteins and mRNAs were further screened by aligning with *FmAG*, *FhAG1*, *FhAG2*, and *FhAG3* published by Zhang et al. (2020), and only the single homolog annotated as *AG* gene from the “Dottato” genome and “Purple Peel” transcriptome was identified and named *FcAG*, while the others were clustered as AGLs. Unfortunately, the recruited AG gene was not assembled into the “Dottato” chromosome.

**FIGURE 1:**
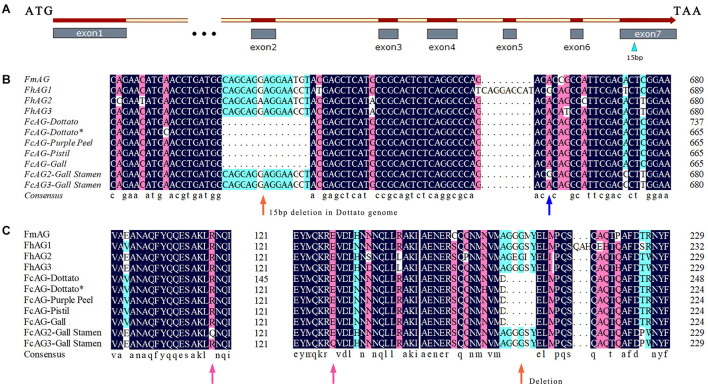
*AG* genes of different *Ficus* species. **(A)** Gene structure of *FcAG*s; the male–female-differentiating 15 bp in the 7th exon is indicated by a blue triangle. **(B)** The 15-bp deletion (orange arrow) is common to the conserved *AG* genes, whereas the 15 bp are present in two other *AG* genes expressed in galls and stamens. Blue arrow indicates base difference. **(C)** Some synonymous and missense mutations (pink arrows) revealed by amino acid sequence prediction of the *AG* genes.

Specific primers AG-F and AG-R were designed between the start and stop coding regions using the *AG* gene of *Ficus* (*F. microcarpa*, *F. hispida*, and *F. carica*) (Table 1). cDNA of “Purple Peel” pistil, and the gall and stamen of male fig cv. Syria_Xu were used as the templates for PCR amplification. Only one *AG* gene was found in the pistils and was named *FcAG-Pistil*. Alignment of *FcAG-Pistil* with the full-length DNA of *FcAG-Dottato* revealed 7 exons (Figure 1A).

Two other *AG* genes were found to be expressed in both stamens and galls, and were named *FcAG2-Gall_Stamen* and *FcAG3-Gall_Stamen*. Another AG homolog specifically expressed in gall tissues was identified and named *FcAG-Gall*, which had only a few base differences from *FcAG-Pistil*. The main difference between *FcAG2-Gall_Stamen*/*FcAG3-Gall_Stamen* and *FcAG-Pistil* was that the two former genes had the 15 bp in the 7th exon that were absent in *FcAG-Dottato*, *FcAG-Purple Peel*, *FcAG-Pistil*, and *FcAG-Gall* (Figures 1A,B). *FcAG2-Gall_Stamen* and *FcAG3-Gall_Stamen* were very similar, with only 9 base differences (Supplementary Figure 1), and the different bases led to changes in two amino acids (Figures 1B,C).

### Clustering Analysis of AGs

**FIGURE 2:**
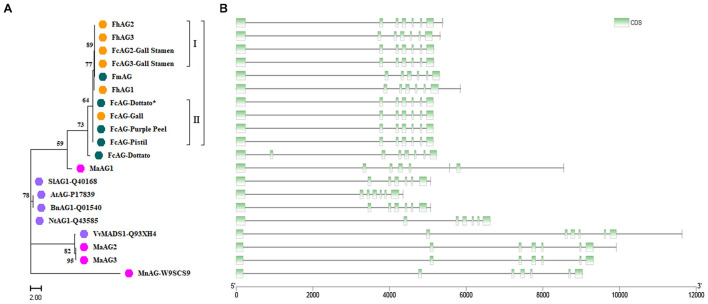
Cluster analysis of *AG* genes and their encoded proteins from various species. **(A)** Phylogenetic tree. Orange – *Ficus* males; green – *Ficus* females; pink – Moraceae plants; purple – other plant species. **(B)** Gene structure. Each gene in panel **B** corresponds to the protein to its left in panel **A**. Green – coding sequence (CDS); black line – intron.

The phylogenetic tree of fig AGs was constructed with the *F. microcarpa* and *F. hispida* counterparts and AtAG (*A. thaliana*, accession number: P17839), BnAG1 (*Brassica napus*, Q01540), NtAG1 (*Nicotiana tabacum*, Q43585), SlAG1 (*Solanum lycopersicum*, Q40168), MnAG (*Morus notabilis*, W9SCS9), and VvMADS1 (*V. vinifera*, Q93XH4) collected from UniProt (Supplementary Table 2). In addition, we recruited three *AG* genes from the *M. alba* genome and translated them into protein sequences. *MaAG2* and *MaAG3* were different transcript variants of the same gene (Figure 2A). The clustering results showed that *Ficus* AG proteins are rather conserved, with the AGs of *F. carica*, *F. microcarpa*, and *F. hispida* being classified into one group. Among this group, FcAG2-Gall_Stamen and FcAG3-Gall_Stamen in male fig clustered closer to the male-specific FhAG2 and FhAG3 of *F. hispida*, and these four AGs composed subgroup I. The remaining AG proteins, except for FmAG and FhAG1, could be classified into subgroup II, sharing the characteristic 15-bp deletion. AGs of the Moraceae family *M. alba* and *M. notabilis* were more clearly distinct from *Ficus* with respect to both protein sequence (Figure 2A) and gene structure (Figure 2B).

There were 7 exons in the *AG* genes of all tested species except *FcAG-Dottato* (Figure 2B). The *FcAG-Dottato* CDS, used for clustering, was extracted from the “Dottato” genome (Usai et al., 2020), where a 71-bp exon insertion was found between the 1st and 2nd exon of other *FcAG*s, and there was a 1-bp insertion in the 3rd exon (Supplementary Figure 1). As the two insertions were not present in the other *Ficus AG*s, we speculated that they were errors in the “Dottato” genome assembly. Therefore, we amplified the *AG* gene from “Dottato” fig fruit cDNA and sequenced it. As predicted, only one *AG* gene was amplified in “Dottato” and the sequence was exactly the same as that of *FcAG-Gall*, *FcAG-Pistil*, and *FcAG-Purple Peel*. We named our cloned and sequenced *AG* gene from “Dottato” *FcAG-Dottato*^∗^, and used it in the nucleotide and amino acid sequence comparisons (Figures 1B,C), the AG phylogenetic tree and gene structure visualization (Figures 2A,B).

### Expression Analysis of AGs in Fig

**FIGURE 3:**
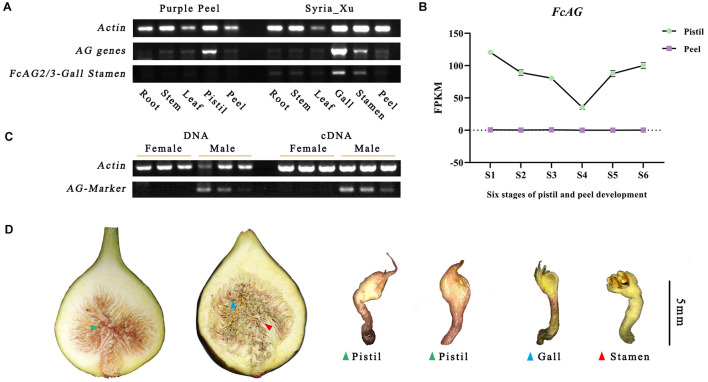
Specific expression of *AG* genes revealed by semi-quantitative RT-PCR and fig fruit transcriptome. **(A)** *AG* genes are widely expressed in fig flowers, but male-specific *AG* genes *FcAG2/3-Gall_Stamen* are only found in fig galls and stamens. **(B)** Transcriptome data of “Purple Peel” fruit reveal that the *FcAG* gene is only expressed in the pistils; no expression was detected in the peel. **(C)** AG-Marker PCR products are only identified in male individuals; both DNA and cDNA can be used as the template. **(D)** Sectional view of a female fig fruit (left) and male fig fruit (right). Both were confirmed by our sex-identification markers.

*FcAG* demonstrated spatial and temporal patterns of expression. Highest expression was found in the gall, followed by the pistil and stamen, and low or no expression in the stem, leaf, peel, and roots by semi-quantitative RT-PCR. *FcAG2-Gall_Stamen* and *FcAG3-Gall_Stamen* transcripts were only detected in the male plants. The gall exhibited higher expression than the stamen, and a very light, if any, band was detected in the other tissues of the male fig plant (Figure 3A).

The same expression pattern was obtained from the transcriptome data of “Purple Peel,” where *FcAG* was only expressed in the pistil, and not in the peel. Along fig fruit development, *FcAG* expression in the pistils decreased when the young fruit formed, with the lowest expression just before the start of ripening, at the S4 stage, then its transcript abundance increased again (Figure 3B).

### Agamous-Marker for Sex Identification by Amplification

We tested the *F. hispida* sex-specific primers designed by Zhang et al. (2020) for amplification of the DNA and cDNA of fig fruit (three females and three males) from six cultivars (Figure 3D and Supplementary Table 1). The primers were not found suitable for sex identification of *F. carica*, and no male-specific fragment was obtained (Supplementary Figure 2).

As *FcAG2-Gall_Stamen* and *FcAG3-Gall_Stamen* were only found in male fig trees, and they both possessed the 15-bp signature sequence that was absent in *FcAG*, the 15-bp sequence was used as the forward primer (AG-Marker-F, Table 1), and 20 bp before the termination site were used as the reverse primer (AG-Marker-R). Using the DNA and cDNA templates of six fig fruit from three male and three female cultivars, amplification resulted in a specific 146-bp fragment in male figs, but not in female figs (Figure 3C), thus establishing the AG-Marker for fig sex determination.

Accurate differentiation of male and female fig plants by the AG-Marker was validated using leaf DNA of 42 fig cultivars (25 male and 17 female) (Supplementary Table 1). The AG-Marker was also used to screen our two F1 populations; 110 female and 132 male trees (1:1.2) were identified in population 1; 49 female and 36 male trees (1:0.73) were identified in population 2 (Supplementary Figure 3 and Supplementary Table 1). In the 3rd year after sowing, 20.25 and 22.35% of the population 1 and 2, respectively, produced flowers. All these flowering trees were female and actually had been identified as female seedlings using the AG-marker. The result of analyzing F1 populations further verified the effectiveness of the AG-Marker. Moreover, it should be noted that application of the AG-Marker did not require the steps of PCR fragment recovery, enzyme digestion and gel verification of the CAPS method, and avoided the possibility of false-positive results after *Hpy*CH4IV digestion.

### *FcRAN1* Gene Structure and Expression Analysis

**FIGURE 4:**
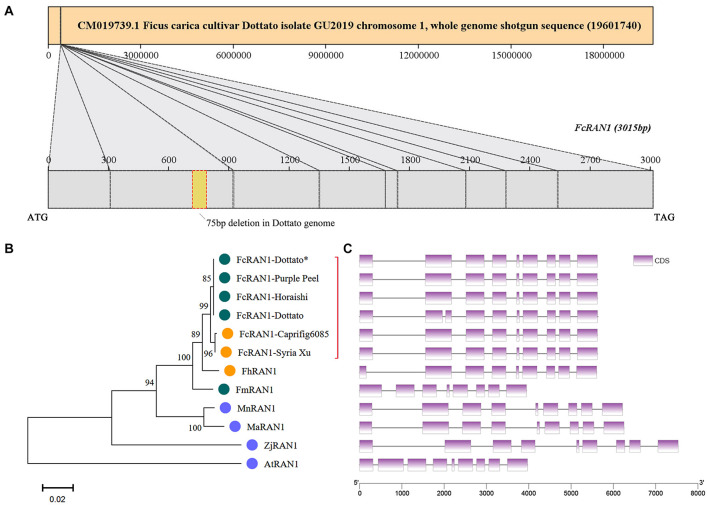
Chromosome localization of *FcRAN1* and clustering of *RAN1* from several species. **(A)** Position of *FcRAN1* on the chromosome and the fragment-deletion annotation error in the published “Dottato” genome. **(B)** Clustering of *RAN1*-encoded proteins of various species. Red bracket highlights close clustering of RAN1s from different fig cultivars. **(C)** Gene structure of *RAN1* in several species. Each gene in panel **C** corresponds to the protein to its left in panel **B**.

The *FcRAN1* sequence of female cv. Purple Peel (ID: *c40459_g1_i1*) was recruited by using TBtools to blast the *FcRAN1* protein-coding sequences of female fig “Horaishi” and male fig “Caprifig6085” (Mori et al., 2017) against our previous “Purple Peel” fruit transcriptome data. The protein-coding sequence of *FcRAN1-Purple Peel* was predicted to be 3015 bp, consistent with the PCR amplification and sequencing results obtained from the cDNA template of “Purple Peel.” *FcRAN1-Purple Peel* was aligned with *FcRAN1-Horaishi* and had identical sequences, both with 9 exons. However, the protein-coding sequence of *FcRAN1-Dottato* (ID: *FCD_00026676*, Supplementary Table 2), extracted from the “Dottato” genome (Usai et al., 2020), was different from that of “Horaishi” and “Purple Peel,” with a 75-bp deletion in the 2nd exon for a *FcRAN1-Dottato* protein-coding sequence of 2940 bp, which was located on Chr01 (Figure 4A). We speculated that the deletion in *FcRAN1-Dottato* might be due to genome assembly error. Therefore, we amplified and sequenced *FcRAN1-Dottato^∗^* from the cDNA of “Dottato” leaves and confirmed that the full-length protein-coding sequence was 3015 bp with 9 exons, like that of “Purple Peel” and “Horaishi.”

A phylogenetic tree was constructed with the protein sequences of RAN1 from fig and other plants, i.e., FmRAN1 (*F. microcarpa*, Gene ID: *Fm.01G0001630*), FhRAN1 (*F. hispida*, *Fh.01G0000580*), MaRAN1 (*M. alba*, *XM_010089630.2*), AtRAN1 (*A. thaliana*, Q9S7J8), ZjRAN1 (*Ziziphus jujuba*, A0A6P4BE01), and MnRAN1 (*M. notabilis*, A0A6P4BE01) (Supplementary Table 2 and Supplementary Figure 4). Clustering results demonstrated that the FcRAN1 protein was rather conserved in *F. carica* and could be divided into two groups: FcRAN1 from the female cultivar and that from the male cultivar (green and orange in Figure 4B, respectively). We used all FcRAN1 CDSs to match the “Dottato” reference genome and there was no significant difference in the exon structure of the *FcRAN1* genes among different fig cultivars (Figure 4C). The gene structure of *FhRAN1* was similar to that of *FcRAN1* except for exon 1, whereas *FmRAN1* was markedly shorter and had 8 exons instead of the 9 exons found in all other *RAN1* genes checked in the present study.

**FIGURE 5:**
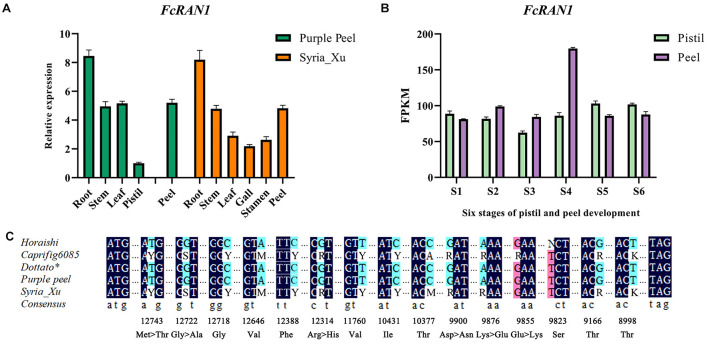
Expression analysis of *FcRAN1* and its SNPs. **(A)** Results of qRT-PCR for *FcRAN1* expression in different tissues of “Purple Peel” (female) and “Syria_Xu” (male) fig trees. **(B)** *FcRAN1* in the transcriptome data of “Purple Peel” fruit. **(C)** Preliminary verification of 15 SNP sites, of which sites seq000259_10377, seq000259_9855, and seq000259_9823 were considered invalid. Degenerate bases: Y, C/T; S, G/C; M, A/C; R, A/G; K, G/T; N, base absence. Dark blue indicates identical bases in both males and females, light blue marks true SNP and pink indicates false SNP.

qRT-PCR analysis revealed wide expression of *FcRAN1* in the roots, stems, leaves, flower, and peel of the female fig cv. Purple Peel and male fig cv. Syria_Xu (Figure 5A). Highest expression of *FcRAN1* was in the roots, similar to *F. hispida* (Zhang et al., 2020). This result could be related to the roots’ prominent role in copper ion absorption and transport. Data mining of the “Purple Peel” fruit transcriptome showed that *FcRAN1* is continuously expressed in the developing pistils and peel, and its expression in peels at the S4 stage was markedly higher than at the other stages (Figure 5B).

Male fig *FcRAN1* was cloned from the leaf cDNA of cv. Syria_Xu. The sequence was aligned with *FcRAN1-Horaishi*, *FcRAN1-Caprifig6085*, *FcRAN1-Purple Peel*, and *FcRAN1-Dottato^∗^*. Multiple SNP sites were identified in the male fig *FcRAN1* (Figure 5C). Among them, there were missense mutations at seq000259_9855, seq000259_9876, seq000259_9900, seq000259_12314, seq000259_12722, and seq000259_12743 sites. The mutations included a transition between positive and negative charges, such as 9855 and 9876; substitution of amino acids with the same chemical properties, such as 12314; change in polarity and non-polarity of amino acids resulting in changes in side-chain hydrophobicity, such as 12722 and 12743. Synonymous mutations were also identified at seq000259_8998, seq000259_9166, seq000259_9823, seq000259_10377, seq000259_10431, seq000259_11760, seq000259_12388, seq000259_12646, and seq000259_12722 sites, which did not change the properties of the amino acids (Supplementary Figure 4).

### Improved Sex-Identification Method Using *RAN1*

**FIGURE 6:**
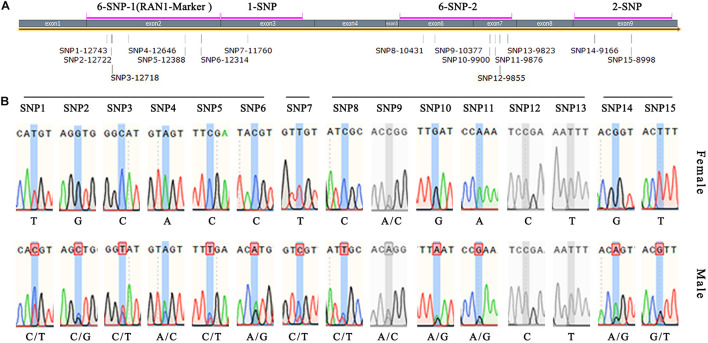
Verification of *FcRAN1* SNP-specific primers. **(A)** Amplification interval of the SNP identification primers and number of covered SNP sites. **(B)** Sex-identification capacity of 15 SNP sites validated by 51 fig cultivars of known sex. Gray color indicates invalid SNPs.

Four pairs of primers (6-SNP-1, 1-SNP, 6-SNP-2, and 2-SNP) were designed to detect the 15 exon-located SNPs in *FcRAN1* (Table 1 and Figure 6A). Specific fragments were amplified and sequenced using 51 known-sex fig cultivars (24 female and 27 male). SNP13-9823, SNP12-9855, and SNP9-10377 were found to not be sex-specific (Figure 6B). In addition to SNP15-8998, SNP11-9876, and SNP6-12314, which were reported by Mori et al. (2017), 9 new SNPs were found with stable sex polymorphism (Figure 6B). In other words, heterozygous double-base peaks characterized the males, while a homozygous single-base peak tagged the females.

Because 6 out of 12 sex-discriminating SNPs were located in the 2nd exon, the 6-SNP-1 primers whose 608-bp amplicon covered these 6 SNPs were renamed RAN1-Marker (Figure 6A). The advantage of this primer pair was that the amplified fragment was located completely within the exon, and therefore the PCR template could be either cDNA or DNA, making it an ideal and stable molecular marker.

The effectiveness of RAN1-Marker was further verified using seedlings from the two F1 populations. In population 1, 110 and 132 seedlings were identified as female and male, respectively, and in population 2, 49 and 36 seedlings were identified as female and male, respectively (Supplementary Table 1). The results were identical to those using the AG-Marker, proving that both methods are equally accurate for fig plant sex identification. However, the AG-Marker method was more convenient due to its one-step PCR compared to our RAN1-Marker-based method, which was nevertheless improved.

### Chromosomal Localization of AGs and *RAN1*

*FmAG* and *FhAG1* were located on their chromosome (Chr) 01 by Zhang et al. (2020); all *F. microcarpa* and *F. hispida* plants have this Chr01 *AG*, which we refer to as the conserved *AG* gene. Our study revealed that the conserved *AG* is also present in female and male fig trees (*FcAG-Pistil* and *FcAG-Gall*). In the “Dottato” genome, *FcAG-Dottato* was annotated but not localized to any chromosome.

In addition to the conserved *AG* gene, there were two other *AG* genes (*FhAG2* and *FhAG3*) in the *F. hispida* genome which were highly homologous with *FcAG2-Gall_Stamen* and *FcAG3-Gall_Stamen* of male fig trees (Figure 2A). *FhAG2* was located on Chr12 and *FhAG3* was not localized to any chromosome. *FhAG2* and *FhAG3* are regarded as two different *AG* genes (Zhang et al., 2020). The *FcAG2-Gall_Stamen* and *FcAG3-Gall_Stamen* genes, which we cloned from the male fig trees, differed by only a few bases, and were likely to be a pair of alleles (Supplementary Figure 1).

**FIGURE 7:**
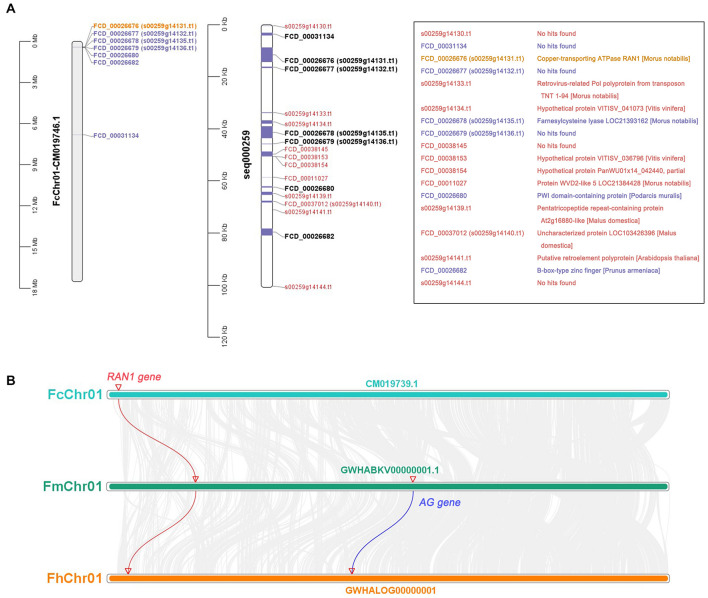
Gene annotation of 0–100 kb in seq000259 scaffold and chromosome collinearity analysis. **(A)** Position of genes on FcChr01 of cv. Dottato (left) and seq000259 scaffold of cv. Horaishi (middle). Orange, purple, and black genes can be matched to Chr01; red genes lack chromosome location information. Gene annotation information is shown in the box (right). **(B)** Chromosome collinearity of *RAN1* and *AG* from three *Ficus* species (*F. carica*, *F. microcarpa*, and *F. hispida*).

The CDSs of protein-coding genes were extracted from the “Dottato” reference genome using TBtools and the gene-annotation file (Supplementary Table 4). They were then aligned with the 100-kb sex-linkage region of the seq000259 scaffold reported by Mori et al. (2017) (Figure 7A middle and right). Twelve protein-coding genes were screened out—seven mapped to FcChr01 (Figure 7A left and middle), and five were not assembled due to lack of chromosome annotation. Six genes were found in the 401567- to 473625-bp region of FcChr01, distanced from *FCD_ 00031134*. Among the six genes, we confirmed that *FcRAN1* (*FCD_00026676* or *s00259g14131.t1*) had accurate sex-discriminating capability. Two genes were not previously reported: *FCD_ 00026682* was annotated as a B-box-type zinc finger protein in the “Dottato” genome, and *FCD_00026680* was similar to the PWI domain-containing protein and *At2g29210* of *Arabidopsis* in UniProt. Results suggested that *FcRAN1* is very likely involved in fig sex determination in this region.

We also checked the genomes of *F. microcarpa* and *F. hispida* and found both *FmRAN1* and *FhRAN1* to be located on Chr01 (Figure 7B). The colocalization results for the conserved *AG* and *RAN1* genes in both *F. microcarpa* and *F. hispida* indicated that the *FcAG-Dottato* gene is probably located on FcChr01 as well. In a recent report, *MaRAN1* and *MaAG1* were also localized to the same chromosome (MaChr04) in *M. alba* genome-annotation files (Jiao et al., 2020).

## Discussion

Sex identification of economically valuable dioecious plants is as important as in animal husbandry. In practice, only female fig trees are vegetatively propagated and grown for fig production. As pollination is required by some types of female fig cultivars, and has a positive effect on fruit quality and storability, selected male fig cultivars are used to provide pollen (Lama et al., 2020; Marcotuli et al., 2020). Sex identification and understanding the mechanism of sex evolution of fig trees are of obvious value to breeding and crop improvement. Therefore, the establishment of a simple and effective method for sex identification of fig seedlings will significantly benefit fig breeding.

### Agamous Genes in Fig Sex Determination

Floral organs directly reflect the sex of plants. In the ABCDE model of plant floral organ formation, the C-type functional AG of the MADS-box family is involved in the formation and differentiation of carpels, stamens, and floral meristems (Dreni and Kater, 2014). In addition, AG subfamily members constitute the D-type functional genes of the MADS-box transcriptional factors, which regulate ovule development. In grape, a missense mutation of one amino acid in VviAGL11 leads to a seedless phenotype (Royo et al., 2018).

In our study, a conserved *AG* gene was found in both male and female plants, i.e., *FcAG-Gall* expressed in the gall of male tree syconia and *FcAG-Pistil* in the pistil of female tree syconia. Previous reports have shown that *AG* is the critical gene regulating both carpel and stamen development (Wellmer et al., 2004; Serwatowska et al., 2014). We found that the conserved *FcAG* has higher expression in the early and late stages of fig syconium development, possibly related to carpel differentiation and maturation (Figure 3B). In *Arabidopsis*, *AG* regulates the expression of *DAD1*, and subsequently promotes the massive synthesis of jasmonic acid that results in anther development (Ito et al., 2007; Jibran et al., 2017). Coexpression of the conserved *AG* and the male-specific *AG* may result in the presence of both male and female flowers in the syconia growing on male trees, namely stamens and galls.

A recent publication recruited two fig *AG* genes (seq00824 and seq00026) by blasting *MnAG* against the “Horaishi” draft genome. Expression of both genes was reported to be much higher in breba (spring fruit) than in the main crop (autumn fruit) (Marcotuli et al., 2020). However, only one conserved *AG* gene was found in our study. Due to the genetic distance between *M. notabilis* and *F. carica* and the revealed phylogenetic difference between *FcAG*s and the mulberry *AG*s (Figure 2A), there could be errors when using *MnAG* to search for *FcAG*. We therefore blasted the “Horaishi” genome using the CDS of *FcAG-Dottato*^∗^, and recruited two genes, seq000824 and seq004379. When they were compared with the gDNA sequence of *FcAG-Dottato*, they were found to cover exon 2 to exon 7, and exon 1 of the conserved *AG*, respectively.

Using the published mulberry (*M. alba*) genome (Jiao et al., 2020), we found three *AG* genes, *MaAG1* (ID: XM_024164781.1-0), *MaAG2* (ID: XM_024174096.1-0), and *MaAG3* (ID: XM 010111053.2-0). Gene structure comparison revealed bigger differences between *MaAG1* and *MaAG2/MaAG3* than between *MaAG2* and *MaAG3*, the latter two found to be two different transcripts of the same gene (*M. alba*_G0002364). Both *MaAG1* and *MaRAN1* genes were located on MaChr04. We speculate that *MaAG1* is the conserved *AG* gene, like *FmAG* and *FhAG1*.

### *RAN1* Could Be Involved in Fig Sex Determination

*RAN1* was cloned and found to encode a copper-transporting P-type ATPase, which belongs to the P1B-Type Heavy Metal ATPase family (Colangelo and Guerinot, 2006). RAN1 is involved in transporting copper ions from the cytoplasm to the Golgi apparatus and plays an essential role in the biogenesis and activation of the ethylene receptors, ETR1 (ETHYLENE RESISTANCE 1), ERS1 (ETHYLENE RESPONSE SENSOR 1), ETR2, EIN4 (ETHYLENE INSENSITIVE 4), and ERS2, and in maintaining copper homeostasis in *Arabidopsis* seedlings (Binder et al., 2010). With an early requirement for *RAN1* in the ethylene pathway, mutation of this gene, which hindered ethylene-binding activity in ethylene signal transduction, gave a phenotype with disrupted development and ripening (Hirayama et al., 1999).

The hormone ethylene regulates floral organ development. When the ethylene signal-transduction process is activated, expression of *ACS11* relieves the inhibitory effect of *WIP1* on *ACS2* (Martin et al., 2009; Hu et al., 2017). *ACS2* promotes ethylene synthesis through positive feedback, increases cellular ethylene levels, promotes pistil formation and inhibits stamen development (Boualem et al., 2008). This regulation mechanism suggests that fine regulation of ethylene level could lead to the formation of all female flowers. Nevertheless, the mechanism underlying the discriminatory capacity of *RAN1* SNPs has yet to be elucidated, and further gene-function studies are required to provide more clues.

### Functional Genes in Plant Sex Determination

About 6% of flowering plant species have individuals of separate sexes (Ming et al., 2011), including a number of horticulturally important crops, such as persimmon (*D. lotus*), kiwifruit (*Actinidia chinensis*), wild grape (*V. vinifera* subsp. *sylvestris*), asparagus (*A. officinalis*), date palm (*P. dactylifera*), red bayberry (*Morella rubra*), willow (*Salix viminalis*), and poplar (*Populus trichocarpa*), together with wild strawberry (*Fragaria virginiana*) and papaya (*Carica papaya*), from which hermaphrodite types have been selected for commercial growing. Sex-linked regions of a few dozen to a few hundred kilobase pairs have been positioned on specific chromosomes of dioecious plants, yet little is known about the specific genes determining sex and their evolutionary history (Henry et al., 2018). Findings in recent years have suggested that more than one gene/transcription factor is involved in plant sex determination. Consequently, it is logical to propose the existence of multiple sex-identification markers with genes involved in plant sex determination (Leite Montalvão et al., 2021).

Two sex-determining genes—*Shy Girl* (*SyGI*) and *Friendly Boy* (*FrBy*)—have been identified from kiwifruit. *SyGI* negatively regulates cytokinin signaling. *FrBy* belongs to the MTR1 family, which contributes to tapetum degradation *via* programmed cell death (Akagi et al., 2014). In asparagus, two independent sex-determining genes, *SOFF* and *aspTDF*, were both localized to Chr5 (Moreno et al., 2018). *TDF1* was found to be a R2R3 MYB transcription factor, in which a single nucleotide mutation leads to male sterility (Harkess et al., 2020). In *V. vinifera* subsp. *sylvestris*, the sex-determination region was located on Chr2, and traces of purifying selection were found with a trehalose phosphatase, an exostosin and a WRKY transcription factor in male alleles (Picq et al., 2014).

Eleven sex-associated markers have been identified in red bayberry, six of them located in a sex-determining region. Using the female phenotype locus W, a homozygous “super female” was generated that produces all female red bayberry offspring in the F2 generation (Wang et al., 2020). Edible fig (*F. carica*) and some other species of *Ficus* are dioecious; the female plants only produce female flowers in the syconia, suggesting repression of male-determining genes. *AG* and *RAN1* both had the capacity to differentiate between males and females, but their biological functions in sex determination have not been validated, and the existence of other markers cannot be excluded.

## Conclusion

The identification of male and female individuals of dioecious plants is of great value to agriculture, the environment and crop breeding. In the present study, one *AG* gene was identified in the female fig tree which was specifically expressed in the pistil. Three *AG* genes were found in the male fig: *FcAG-Gall* was specifically expressed in the gall, whereas *FcAG2-Gall_Stamen* and *FcAG3-Gall_Stamen* were expressed in the gall and stamen. A highly efficient AG-Marker-based method was successfully established for fig sex identification, and the previously reported RAN1-Marker method was improved. Our results provide new markers and new clues to the sex-determination system of *F. carica*.

## Data Availability Statement

The original contributions presented in the study are included in the article/Supplementary Material, further inquiries can be directed to the corresponding author.

## Author Contributions

XW and HM designed the experiments. XW and MS conducted the experiments and analyzed the results. HM, MF, and SC revised the manuscript. All authors prepared, read, and approved the manuscript for publication.

## Conflict of Interest

The authors declare that the research was conducted in the absence of any commercial or financial relationships that could be construed as a potential conflict of interest.

## Publisher’s Note

All claims expressed in this article are solely those of the authors and do not necessarily represent those of their affiliated organizations, or those of the publisher, the editors and the reviewers. Any product that may be evaluated in this article, or claim that may be made by its manufacturer, is not guaranteed or endorsed by the publisher.

## Footnotes

1
https://blast.ncbi.nlm.nih.gov/Blast.cgi

2
https://www.uniprot.org/blast/

3
https://www.ncbi.nlm.nih.gov/orffinder/

4
https://www.novopro.cn/tools/translate.html

5
https://www.megasoftware.net/

6
https://www.lynnon.com/

7
https://www.snapgene.com/try-snapgene/

8
https://ngdc.cncb.ac.cn/

## Supplementary Material

Supplementary Figure 1 Full-length alignment of *Ficus AG* genes.

Supplementary Figure 2 PCR amplification results of *Ficus hispida* sex-specific primers using *Ficus carica* DNA as the template.

Supplementary Figure 3 AG-Marker validation results for two F1 populations. “M” marks male individuals and “F” marks female individuals.

Supplementary Figure 4 Alignment of *RAN1* amino acid sequences from different species. Blue box indicates SNP site.

Supplementary Table 1 List of fig cultivars and sex-identification results. Sheet 1 includes all fig cultivars and populations tested in this study. Light green marks female and light orange marks male. Sheet 2 presents the statistical data pertaining to the results.

Supplementary Table 2 Coding sequences (CDS) and protein sequences used in this study.

Supplementary Table 3 AG-related sequences screened from “Purple Peel” fig transcriptome and “Dottato” fig protein files. Red bold, the only *AG* gene screened.

Supplementary Table 4 Protein-coding gene sequences extracted from the “Dottato” genome.
