# Supplementary material for: AGAMOUS Gene as a New Sex-Identification Marker in Fig (Ficus carica L.) Is More Efficient Than RAN1
Source: Front Plant Sci. 2021 Oct 20;12:755358. doi: 10.3389/fpls.2021.755358 (PMC8564383; doi:10.3389/fpls.2021.755358)
Supplement: Supplementary Figure 1 — Full-length alignment of Ficus AG genes. [file Data_Sheet_1.zip › Supplementary Table 2.DOCX]

**Supplementary Table 2** CDS and protein sequences in this study.

**Contents**

[CDS sequences 3](#_Toc74244259)

[>FcRAN1-Horaishi 3](#_Toc74244260)

[>FcRAN1-Caprifig6085 4](#_Toc74244261)

[>FcRAN1-Dottato 5](#_Toc74244262)

[>FcRAN1-Dottato* 7](#_Toc74244263)

[>FcRAN1-Purple Peel 8](#_Toc74244264)

[>FcRAN1-Syrai_Xu 10](#_Toc74244265)

[>FhRAN1 11](#_Toc74244266)

[>FmRAN1 12](#_Toc74244267)

[>MaRAN1-XM_010089630.2 14](#_Toc74244268)

[>FmAG 15](#_Toc74244269)

[>FhAG1 16](#_Toc74244270)

[>FhAG2 16](#_Toc74244271)

[>FhAG3 17](#_Toc74244272)

[>FcAG-Dottato 17](#_Toc74244273)

[>FcAG-Dottato* 17](#_Toc74244274)

[>FcAG-Pistil 18](#_Toc74244275)

[>FcAG-Purple Peel 18](#_Toc74244276)

[>FcAG-Gall 19](#_Toc74244277)

[>FcAG2-Gall 19](#_Toc74244278)

[>FcAG3-Gall 19](#_Toc74244279)

[>FcAG2-Stamen 20](#_Toc74244280)

[>FcAG3-Stamen 20](#_Toc74244281)

[>MaAG1(XM_024164781.1-0) 21](#_Toc74244282)

[>MaAG2(XM_024174096.1-0) 21](#_Toc74244283)

[>MaAG3(XM_010111053.2-0) 21](#_Toc74244284)

[Protein sequence 22](#_Toc74244285)

[>AtRAN1-Q9S7J8 22](#_Toc74244286)

[>MnRAN1-W9SDE0 23](#_Toc74244287)

[>ZjRAN1-A0A6P4BE01 23](#_Toc74244288)

[>AtAG-P17839 24](#_Toc74244289)

[>BnAG1-Q01540 24](#_Toc74244290)

[>NtAG1-Q43585 25](#_Toc74244291)

[>SlAG1-Q40168 25](#_Toc74244292)

[>VvMADS1-Q93XH4 25](#_Toc74244293)

# CDS sequences

## >FcRAN1-Horaishi

atggcggcgagcgtccgacacctCCAGCTCACCCAACTCTCCGCCGCCGGCGCCGGCGACGACGACGACTCCGGCGACCTCGAGGACGTGCGGCTCCTCGACGCGTACAAGAATTCGGAGGAGAACGATGAGGGAGTTGTCGGAGAGGCGACGATGAAGAGGATTCAGGTCGGCGTCACCGGCATGACCTGCGCGGCTTGCTCGAACTCCGTCGAAGCTGCTCTGATGAGCGTCAATGGCGTTCTCAGGGCTTCCGTCGCTCTGCTCCAGAACAAGGCCGACGTGGTCTTCGATCCCAGATTGGTCAAGGATGAAGATATCAAGAATGCAATCGAGGATGCTGGGTTTGAAGCTGAGATTCTACCTGAATCAAGTGCCATTGGAACAAAGCCTCAAGCGACCATGTTAGGGCAGTTCTCTATAGGTGGCATGACATGTGCGGCTTGTGTGAACTCGGTAGAAGGCATTTTACGAGATCTTCCCGGTGTCAAAAGGGCTGTAGTTGCCTTGGCTACTTCATTAGGTGAAGTTGAGTATGATCCAGCTGTTATCAGTAAAGAGGATATCGTCAATGCAATTGAAGACGCTGGTTTTGAAGGAGCCTTTGTACAGAGCAGTGAACAAGATAAGATTGTGTTGGGCGTTGCTGGCATATACAGTGAGATGGATGTACAGATTTTAGGAGGCATACTTAGCAACTTGAAAGGGGTGAGACAATTTCATTTTGACCGAATTTCAAGAGAACTTGAAGTTCTGTTCGATCCTGAAGTTATTCATTCAAGATCCTTAGTTGATGGGGTTGAAGGGGGAAGTAGCGGAAGGTTTAAATTACGTGTTGCTAATCCTTATTCAAGAATGACTTCTAAAGATGTGGAAGAAGCCTCAAACATGTTTAGACTGTTCATCTCTAGTCTATTTCTCAGCGTTCCTGTCTTTCTCATACGAGTAGTTTGTCCACACATACCACTGATTTATTCCTTATTGCTTTGGCGATGTGGGCCCTTTCAAATGGGTGACTGGTTGAAGTGGGCGTTGGTGAGTGTTGTTCAATTTGTTGTTGGAAAGCGCTTCTACATTGCTGCTGCAAGAGCTCTACGAAATGGTTCAACTAACATGGATGTTTTGGTTGCATTGGGGACATCAGCCTCCTATTTCTACTCTGTTTGTGCACTTCTATATGGTGCACTCACTGGGTTTTGGTCCCCAACTTACTTTGAAACAAGTGCAATGCTGATTACATTTGTGCTGTTAGGAAAATATCTAGAGTGTCTTGCAAAGGGGAAAACATCTGACGCCATCAAAAAGTTGGTAGAACTTGCACCAGCAACGGCAACATTGCTTATCAAGGATAAAGATGGAAGATATCTTGGAGAAAGGGAAATAGATGCTCTACTAATTCAGCCCGGTGACACATTGAAAGTTTCACCGGGTGCAAAATTACCTGCTGATGGTGTTGTTGCTTGGGGTTCAAGTTATGTAAATGAAAGTATGGTAACTGGTGAATCTGTACCCATTTCGAAGGAGGTCGGTTCATCAGTAATTGGAGGTACAATAAATTTACATGGTGCTCTACACGTACAAGCTACCAAAGTAGGTTCTGACACAGTTTTGAGCCAGATAATCAGTTTAGTTGAAACAGCGCAGATGTCCAAAGCTCCTATTCAGAAATTTGCTGATTTCATCGCTAGCATTTTTGTTCCTACAGTTGTTTTGTTGGCATTGTTGACGTTATTAGGATGGTACGCTGCTGGAGCTCTTCGTGCTTACCCAGAAAGTTGGCTTCCAGAAAATGGAAATTACTTTGTTTTCGCCCTTATGTTTTCTATATCAGTTGTGGTGATCGCGTGCCCCTGTGCTCTTGGCTTGGCAACACCAACTGCGGTCATGGTTGCAACCGGAGTCGGTGCTAACAATGGTGTGCTGATAAAAGGGGGCGATGCCTTAGAAAGGGCTCAAAAAATAAAGTATGTGATCTTTGATAAAACAGGTACCCTAACTCAGGGAAAGGCCTCGGTTACAACTACAAAAGTGTTTTCGGGAATGGATCGTGGAGAATTCCTCAAGTTGGTGGCTTCTGCTGAGGCTAGTAGTGAACATCCACTGGCAAAGGCAATAGTTGCATATGCCCGTCATTTTCATTTCTTTGATGATTCTGCTACCAAGGATGCCAAAAGCCATAGCAAAGACTCCGAAGTATCTGGTTGGCTTTTTGATGTAGCAGAATTTNCTACTCTTCCCGGCAGAGGAGTTCAATGCTTTATTAATGAAAAACAGATTTTGGTTGGTAATCGCAAGTTGATGACCGAAAGTGGAATCGACATCCCTGATGATGTAGAAAAATTTGTTGTAGAACTCGAAGAAAGTGCGAAAACAGGCATACTTGTCGCATATAACGGTAACTTAATTGGTGTTTTGGGGGTTGCAGACCCACTGAAAAGAGAGGCAGCTGTGGTAGTTGAGGGCCTGAGTAAAATGGGCATCCGACCGGTCATGGTTACTGGGGACAACTGGAGGACAGCGCAGGCTGTTGCTAAGGAGGTTGGCATTCACGATGTAAGGGCAGAGGTAATGCCTGCAGGAAAAGCCGATGTAGTTCGTTCGTTTCATAAGGATGGAATCACGGTTGCGATGGTGGGAGACGGAATCAATGATTCTCCAGCTCTAGCTGCAGCAGATGTTGGCATGGCAATTGGAGCAGGGACGGATATTGCCATTGAAGCGGCCAACTATGTTTTGATGAGGAACAACTTGGAGGATGTGATCACCGCCATTGATCTCTCAAGAAAGACTTTCTCTCGAATCAGATTAAATTATGTGTTTGCCATGGCTTACAATGTCATAGCAATCCCTGTTGCTGCCGGAGTTTTCTTTCCGTCGTTGGGGATTCAGTTGCCGCCATGGGCGGCTGGTGCATGCATGGCTTTGTCTTCTGTTAGTGTTGTATGTTCTTCTCTATTACTTAGGAGATACAGAAAACCAAGgctgaccactatactagaaataaCTGTAGAATAG

## >FcRAN1-Caprifig6085

atggcggcgagcgtccgacacctCCAGCTCACCCAACTCTCCGCCGCCGGCGCCGGCGACGACGACGACTCCGGCGACCTCGAGGACGTGCGGCTCCTCGACGCGTACAAGAATTCGGAGGAGAACGATGAGGGAGTTGTCGGAGAGGCGACGATGAAGAGGATTCAGGTCGGCGTCACCGGCATGACCTGCGCGGCTTGCTCGAACTCCGTCGAAGCTGCTCTGATGAGCGTCAATGGCGTTCTCAGGGCTTCCGTCGCTCTGCTCCAGAACAAGGCCGACGTGGTCTTCGATCCCAGATTGGTCAAGGATGAAGATATCAAGAATGCAATCGAGGATGCTGGGTTTGAAGCTGAGATTCTACCTGAATCAAGTGCCATTGGAACAAAGCCTCAAGCGACCACGTTAGGGCAGTTCTCTATAGCTGGTATGACATGTGCGGCTTGTGTGAACTCGGTAGAAGGCATTTTACGAGATCTTCCCGGTGTCAAAAGGGCTGTCGTTGCCTTGGCTACTTCATTAGGTGAAGTTGAGTATGATCCAGCTGTTATCAGTAAAGAGGATATCGTCAATGCAATTGAAGACGCTGGTTTTGAAGGAGCCTTTGTACAGAGCAGTGAACAAGATAAGATTGTGTTGGGCGTTGCTGGCATATACAGTGAGATGGATGTACAGATTTTAGGAGGCATACTTAGCAACTTGAAAGGGGTGAGACAATTTCATTTTGACCGAATTTCAAGAGAACTTGAAGTTCTGTTTGATCCTGAAGTTATTCATTCAAGATCCTTAGTTGATGGGGTTGAAGGGGGAAGTAGCGGAAGGTTTAAATTACATGTTGCTAATCCTTATTCAAGAATGACTTCTAAAGATGTGGAAGAAGCCTCAAACATGTTTAGACTGTTCATCTCTAGTCTATTTCTCAGCGTTCCTGTCTTTCTCATACGAGTAGTTTGTCCACACATACCACTGATTTATTCCTTATTGCTTTGGCGATGTGGGCCCTTTCAAATGGGTGACTGGTTGAAGTGGGCGTTGGTGAGTGTCGTTCAATTTGTTGTTGGAAAGCGCTTCTACATTGCTGCTGCAAGAGCTCTACGAAATGGTTCAACTAACATGGATGTTTTGGTTGCATTGGGGACATCAGCCTCCTATTTCTACTCTGTTTGTGCACTTCTATATGGTGCACTCACTGGGTTTTGGTCCCCAACTTACTTTGAAACAAGTGCAATGCTGATTACATTTGTGCTGTTAGGAAAATATCTAGAGTGTCTTGCAAAGGGGAAAACATCTGACGCCATCAAAAAGTTGGTAGAACTTGCACCAGCAACGGCAACATTGCTTATCAAGGATAAAGATGGAAGATATCTTGGAGAAAGGGAAATAGATGCTCTACTAATTCAGCCCGGTGACACATTGAAAGTTTCACCGGGTGCAAAATTACCTGCTGATGGTGTTGTTGCTTGGGGTTCAAGTTATGTAAATGAAAGTATGGTAACTGGTGAATCTGTACCCATTTCGAAGGAGGTCGGTTCATCAGTAATTGGAGGTACAATAAATTTACATGGTGCTCTACACGTACAAGCTACCAAAGTAGGTTCTGACACAGTTTTGAGCCAGATAATCAGTTTAGTTGAAACAGCGCAGATGTCCAAAGCTCCTATTCAGAAATTTGCTGATTTCATCGCTAGCATTTTTGTTCCTACAGTTGTTTTGTTGGCATTGTTGACGTTATTAGGATGGTACGCTGCTGGAGCTCTTCGTGCTTACCCAGAAAGTTGGCTTCCAGAAAATGGAAATTACTTTGTTTTCGCCCTTATGTTTTCTATATCAGTTGTGGTGATTGCGTGCCCCTGTGCTCTTGGCTTGGCAACACCAACTGCGGTCATGGTTGCAACAGGAGTCGGTGCTAACAATGGTGTGCTGATAAAAGGGGGCGATGCCTTAGAAAGGGCTCAAAAAATAAAGTATGTGATCTTTGATAAAACAGGTACCCTAACTCAGGGAAAGGCCTCGGTTACAACTACAAAAGTGTTTTCGGGAATGGATCGTGGAGAATTCCTCAAGTTGGTGGCTTCTGCTGAGGCTAGTAGTGAACATCCACTGGCAAAGGCAATAGTTGCATATGCCCGTCATTTTCATTTCTTTAATGATTCTGCTACCAAGGATGCCGAAAGCCATAGCAAAGACTCCAAAGTATCTGGTTGGCTTTTTGATGTAGCAGAATTTTCTACTCTTCCCGGCAGAGGAGTTCAATGCTTTATTAATGAAAAACAGATTTTGGTTGGTAATCGCAAGTTGATGACCGAAAGTGGAATCGACATCCCTGATGATGTAGAAAAATTTGTTGTAGAACTCGAAGAAAGTGCGAAAACAGGCATACTTGTCGCATATAACGGTAACTTAATTGGTGTTTTGGGGGTTGCAGACCCACTGAAAAGAGAGGCAGCTGTGGTAGTTGAGGGCCTGAGTAAAATGGGCATCCGACCGGTCATGGTTACTGGGGACAACTGGAGGACAGCGCAGGCTGTTGCTAAGGAGGTTGGCATTCACGATGTAAGGGCAGAGGTAATGCCTGCAGGAAAAGCCGATGTAGTTCGTTCGTTTCATAAGGATGGAATCACAGTTGCGATGGTGGGAGACGGAATCAATGATTCTCCAGCTCTAGCTGCAGCAGATGTTGGCATGGCAATTGGAGCAGGGACGGATATTGCCATTGAAGCGGCCAACTATGTTTTGATGAGGAACAACTTGGAGGATGTGATCACCGCCATTGATCTCTCAAGAAAGACGTTCTCTCGAATCAGATTAAATTATGTGTTTGCCATGGCTTACAATGTCATAGCAATCCCTGTTGCTGCCGGAGTTTTCTTTCCGTCGTTGGGGATTCAGTTGCCGCCATGGGCGGCTGGTGCATGCATGGCTTTGTCTTCTGTTAGTGTTGTATGTTCTTCTCTATTACTTAGGAGATACAGAAAACCAAGgctgaccactatactagaaataaCTGTAGAATAG

## >FcRAN1-Dottato

ATGGCGGCGAGCGTCCGACACCTCCAGCTCACCCAACTCTCCGCCGCCGGCGCCGGCGACGACGACGACTCCGGCGACCTCGAGGACGTGCGGCTCCTCGACGCGTACAAGAATTCGGAGGAGAACGATGAGGGAGTTGTCGGAGAGGCGACGATGAAGAGGATTCAGGTCGGCGTCACCGGCATGACCTGCGCGGCTTGCTCGAACTCCGTCGAAGCTGCTCTGATGAGCGTCAATGGCGTTCTCAGGGCTTCCGTCGCTCTGCTCCAGAACAAGGCCGACGTGGTCTTCGATCCCAGATTGGTCAAGGATGAAGATATCAAGAATGCAATCGAGGATGCTGGGTTTGAAGCTGAGATTCTACCTGAATCAAGTGCCATTGGAACAAAGCCTCAAGCGACCATGTTAGGGCAGTTCTCTATAGGTGGCATGACATGTGCGGCTTGTGTGAACTCGGTAGAAGGCATTTTACGAGATCTTCCCGGTGTCAAAAGGGCTGTAGTTGCCTTGGCTACTTCATTAGGTGAAGTTGAGTATGATCCAGCTGTTATCAGTAAAGAGGATATCGTCAATGCAATTGAAGACGCTGGTTTTGAAGGAGCCTTTGTACAGAGCAGTGAACAAGATAAGATTGTGTTGGGCGTTGCTGGCATATACAGTGAGATGGATGTACAGATTTTAGGAGGCATACTTAGCAACTTGAAAGGATCCTTAGTTGATGGGGTTGAAGGGGGAAGTAGCGGAAGGTTTAAATTACGTGTTGCTAATCCTTATTCAAGAATGACTTCTAAAGATGTGGAAGAAGCCTCAAACATGTTTAGACTGTTCATCTCTAGTCTATTTCTCAGCGTTCCTGTCTTTCTCATACGAGTAGTTTGTCCACACATACCACTGATTTATTCCTTATTGCTTTGGCGATGTGGGCCCTTTCAAATGGGTGACTGGTTGAAGTGGGCGTTGGTGAGTGTTGTTCAATTTGTTGTTGGAAAGCGCTTCTACATTGCTGCTGCAAGAGCTCTACGAAATGGTTCAACTAACATGGATGTTTTGGTTGCATTGGGGACATCAGCCTCCTATTTCTACTCTGTTTGTGCACTTCTATATGGTGCACTCACTGGGTTTTGGTCCCCAACTTACTTTGAAACAAGTGCAATGCTGATTACATTTGTGCTGTTAGGAAAATATCTAGAGTGTCTTGCAAAGGGGAAAACATCTGACGCCATCAAAAAGTTGGTAGAACTTGCACCAGCAACGGCAACATTGCTTATCAAGGATAAAGATGGAAGATATCTTGGAGAAAGGGAAATAGATGCTCTACTAATTCAGCCCGGTGACACATTGAAAGTTTCACCGGGTGCAAAATTACCTGCTGATGGTGTTGTTGCTTGGGGTTCAAGTTATGTAAATGAAAGTATGGTAACTGGTGAATCTGTACCCATTTCGAAGGAGGTCGGTTCATCAGTAATTGGAGGTACAATAAATTTACATGGTGCTCTACACGTACAAGCTACCAAAGTAGGTTCTGACACAGTTTTGAGCCAGATAATCAGTTTAGTTGAAACAGCGCAGATGTCCAAAGCTCCTATTCAGAAATTTGCTGATTTCATCGCTAGCATTTTTGTTCCTACAGTTGTTTTGTTGGCATTGTTGACGTTATTAGGATGGTACGCTGCTGGAGCTCTTCGTGCTTACCCAGAAAGTTGGCTTCCAGAAAATGGAAATTACTTTGTTTTCGCCCTTATGTTTTCTATATCAGTTGTGGTGATCGCGTGCCCCTGTGCTCTTGGCTTGGCAACACCAACTGCGGTCATGGTTGCAACCGGAGTCGGTGCTAACAATGGTGTGCTGATAAAAGGGGGCGATGCCTTAGAAAGGGCTCAAAAAATAAAGTATGTGATCTTTGATAAAACAGGTACCCTAACTCAGGGAAAGGCCTCGGTTACAACTACAAAAGTGTTTTCGGGAATGGATCGTGGAGAATTCCTCAAGTTGGTGGCTTCTGCTGAGGCTAGTAGTGAACATCCACTGGCAAAGGCAATAGTTGCATATGCCCGTCATTTTCATTTCTTTGATGATTCTGCTACCAAGGATGCCAAAAGCCATAGCAAAGACTCCGAAGTATCTGGTTGGCTTTTTGATGTAGCAGAATTTTCTACTCTTCCCGGCAGAGGAGTTCAATGCTTTATTAATGAAAAACAGATTTTGGTTGGTAATCGCAAGTTGATGACCGAAAGTGGAATCGACATCCCTGATGATGTAGAAAAATTTGTTGTAGAACTCGAAGAAAGTGCGAAAACAGGCATACTTGTCGCATATAACGGTAACTTAATTGGTGTTTTGGGGGTTGCAGACCCACTGAAAAGAGAGGCAGCTGTGGTAGTTGAGGGCCTGAGTAAAATGGGCATCCGACCGGTCATGGTTACTGGGGACAACTGGAGGACAGCGCAGGCTGTTGCTAAGGAGGTTGGCATTCACGATGTAAGGGCAGAGGTAATGCCTGCAGGAAAAGCCGATGTAGTTCGTTCGTTTCATAAGGATGGAATCACGGTTGCGATGGTGGGAGACGGAATCAATGATTCTCCAGCTCTAGCTGCAGCAGATGTTGGCATGGCAATTGGAGCAGGGACGGATATTGCCATTGAAGCGGCCAACTATGTTTTGATGAGGAACAACTTGGAGGATGTGATCACCGCCATTGATCTCTCAAGAAAGACTTTCTCTCGAATCAGATTAAATTATGTGTTTGCCATGGCTTACAATGTCATAGCAATCCCTGTTGCTGCCGGAGTTTTCTTTCCGTCGTTGGGGATTCAGTTGCCGCCATGGGCGGCTGGTGCATGCATGGCTTTGTCTTCTGTTAGTGTTGTATGTTCTTCTCTATTACTTAGGAGATACAGAAAACCAAGGCTGACCACTATACTAGAAATAACTGTAGAATAG

## >FcRAN1-Dottato*

**ATG**GCGGCGAGCGTCCGACACCTCCAGCTCACCCAACTCTCCGCCGCCGGCGCCGGCGACGACGACGACTCCGGCGACCTCGAGGACGTGCGGCTCCTCGACGCGTACAAGAATTCGGAGGAGAAC**GAT**GAGGGAGTTGTCGGAGAGGCGACGATGAAGAGGATTCAGGTCGGCGTCACCGGCATGACCTGCGCGGCTTGCTCGAACTCCGTCGAAGCTGCTCTGATGAGCGTCAATGGCGTTCTCAGGGCTTCCGTCGCTCTGCTCCAGAACAAGGCCGACGTGGTCTTCGATCCCAGATTGGTCAAG**GAT**GAAGATATCAAGAATGCAATCGAGGATGCTGGGTTTGAAGCTGAGATTCTACCTGAATCAAGTGCCATTGGAACAAAGCCTCAAGCGACCATGTTAGGGCAGTTCTCTATAGGTGGCATGACATGTGCGGCTTGTGTGAACTCGGTAGAAGGCATTTTACGAGATCTTCCCGGTGTCAAAAGGGCTGTAGTTGCCTTGGCTACTTCATTAGGTGAAGTTGAGTATGATCCAGCTGTTATCAGTAAAGAGGATATCGTCAATGCAATTGAAGACGCTGGTTTTGAAGGAGCCTTTGTACAGAGCAGTGAACAAGATAAGATTGTGTTGGGCGTTGCTGGCATATACAGTGAGATGGATGTACAGATTTTAGGAGGCATACTTAGCAACTTGAAAGGGGTGAGACAATTTCATTTTGACCGAATTTCAAGAGAACTTGAAGTTCTGTTCGATCCTGAAGTTATTCATTCAAGATCCTTAGTTGATGGGGTTGAAGGGGGAAGTAGCGGAAGGTTTAAATTACGTGTTGCTAATCCTTATTCAAGAATGACTTCTAAAGATGTGGAAGAAGCCTCAAACATGTTTAGACTGTTCATCTCTAGTCTATTTCTCAGCGTTCCTGTCTTTCTCATACGAGTAGTTTGTCCACACATACCACTGATTTATTCCTTATTGCTTTGGCGATGTGGGCCCTTTCAAATGGGTGACTGGTTGAAGTGGGCGTTGGTGAGTGTTGTTCAATTTGTTGTTGGAAAGCGCTTCTACATTGCTGCTGCAAGAGCTCTACGAAATGGTTCAACTAACATGGATGTTTTGGTTGCATTGGGGACATCAGCCTCCTATTTCTACTCTGTTTGTGCACTTCTATATGGTGCACTCACTGGGTTTTGGTCCCCAACTTACTTTGAAACAAGTGCAATGCTGATTACATTTGTGCTGTTAGGAAAATATCTAGAGTGTCTTGCAAAGGGGAAAACATCTGACGCCATCAAAAAGTTGGTAGAACTTGCACCAGCAACGGCAACATTGCTTATCAAGGATAAAGATGGAAGATATCTTGGAGAAAGGGAAATAGATGCTCTACTAATTCAGCCCGGTGACACATTGAAAGTTTCACCGGGTGCAAAATTACCTGCTGATGGTGTTGTTGCTTGGGGTTCAAGTTATGTAAATGAAAGTATGGTAACTGGTGAATCTGTACCCATTTCGAAGGAGGTCGGTTCATCAGTAATTGGAGGTACAATAAATTTACATGGTGCTCTACACGTACAAGCTACCAAAGTAGGTTCTGACACAGTTTTGAGCCAGATAATCAGTTTAGTTGAAACAGCGCAGATGTCCAAAGCTCCTATTCAGAAATTTGCTGATTTCATCGCTAGCATTTTTGTTCCTACAGTTGTTTTGTTGGCATTGTTGACGTTATTAGGATGGTACGCTGCTGGAGCTCTTCGTGCTTACCCAGAAAGTTGGCTTCCAGAAAATGGAAATTACTTTGTTTTCGCCCTTATGTTTTCTATATCAGTTGTGGTGATCGCGTGCCCCTGTGCTCTTGGCTTGGCAACACCAACTGCGGTCATGGTTGCAACCGGAGTCGGTGCTAACAATGGTGTGCTGATAAAAGGGGGCGATGCCTTAGAAAGGGCTCAAAAAATAAAGTATGTGATCTTTGATAAAACAGGTACCCTAACTCAGGGAAAGGCCTCGGTTACAACTACAAAAGTGTTTTCGGGAATGGATCGTGGAGAATTCCTCAAGTTGGTGGCTTCTGCTGAGGCTAGTAGTGAACATCCACTGGCAAAGGCAATAGTTGCATATGCCCGTCATTTTCATTTCTTTGATGATTCTGCTACCAAGGATGCCAAAAGCCATAGCAAAGACTCCGAAGTATCTGGTTGGCTTTTTGATGTAGCAGAATTTTCTACTCTTCCCGGCAGAGGAGTTCAATGCTTTATTAATGAAAAACAGATTTTGGTTGGTAATCGCAAGTTGATGACCGAAAGTGGAATCGACATCCCTGATGATGTAGAAAAATTTGTTGTAGAACTCGAAGAAAGTGCGAAAACAGGCATACTTGTCGCATATAACGGTAACTTAATTGGTGTTTTGGGGGTTGCAGACCCACTGAAAAGAGAGGCAGCTGTGGTAGTTGAGGGCCTGAGTAAAATGGGCATCCGACCGGTCATGGTTACTGGGGACAACTGGAGGACAGCGCAGGCTGTTGCTAAGGAGGTTGGCATTCACGATGTAAGGGCAGAGGTAATGCCTGCAGGAAAAGCCGATGTAGTTCGTTCGTTTCATAAGGATGGAATCACGGTTGCGATGGTGGGAGACGGAATCAATGATTCTCCAGCTCTAGCTGCAGCAGATGTTGGCATGGCAATTGGAGCAGGGACGGATATTGCCATTGAAGCGGCCAACTATGTTTTGATGAGGAACAACTTGGAGGATGTGATCACCGCCATTGATCTCTCAAGAAAGACTTTCTCTCGAATCAGATTAAATTATGTGTTTGCCATGGCTTACAATGTCATAGCAATCCCTGTTGCTGCCGGAGTTTTCTTTCCGTCGTTGGGGATTCAGTTGCCGCCATGGGCGGCTGGTGCATGCATGGCTTTGTCTTCTGTTAGTGTTGTATGTTCTTCTCTATTACTTAGGAGATACAGAAAACCAAGGCTGACCACTATACTAGAAATAACTGTAGAATAGAAAGTTTC

## >FcRAN1-Purple peel

ATGGCGGCGAGCGTCCGACACCTCCAGCTCACCCAACTCTCCGCCGCCGGCGCCGGCGACGACGACGACTCCGGCGACCTCGAGGACGTGCGGCTCCTCGACGCGTACAAGAATTCGGAGGAGAACGATGAGGGAGTTGTCGGAGAGGCGACGATGAAGAGGATTCAGGTCGGCGTCACCGGCATGACCTGCGCGGCTTGCTCGAACTCCGTCGAAGCTGCTCTGATGAGCGTCAATGGCGTTCTCAGGGCTTCCGTCGCTCTGCTCCAGAACAAGGCCGACGTGGTCTTCGATCCCAGATTGGTCAAGGATGAAGATATCAAGAATGCAATCGAGGATGCTGGGTTTGAAGCTGAGATTCTACCTGAATCAAGTGCCATTGGAACAAAGCCTCAAGCGACCATGTTAGGGCAGTTCTCTATAGGTGGCATGACATGTGCGGCTTGTGTGAACTCGGTAGAAGGCATTTTACGAGATCTTCCCGGTGTCAAAAGGGCTGTAGTTGCCTTGGCTACTTCATTAGGTGAAGTTGAGTATGATCCAGCTGTTATCAGTAAAGAGGATATCGTCAATGCAATTGAAGACGCTGGTTTTGAAGGAGCCTTTGTACAGAGCAGTGAACAAGATAAGATTGTGTTGGGCGTTGCTGGCATATACAGTGAGATGGATGTACAGATTTTAGGAGGCATACTTAGCAACTTGAAAGGGGTGAGACAATTTCATTTTGACCGAATTTCAAGAGAACTTGAAGTTCTGTTCGATCCTGAAGTTATTCATTCAAGATCCTTAGTTGATGGGGTTGAAGGGGGAAGTAGCGGAAGGTTTAAATTACGTGTTGCTAATCCTTATTCAAGAATGACTTCTAAAGATGTGGAAGAAGCCTCAAACATGTTTAGACTGTTCATCTCTAGTCTATTTCTCAGCGTTCCTGTCTTTCTCATACGAGTAGTTTGTCCACACATACCACTGATTTATTCCTTATTGCTTTGGCGATGTGGGCCCTTTCAAATGGGTGACTGGTTGAAGTGGGCGTTGGTGAGTGTTGTTCAATTTGTTGTTGGAAAGCGCTTCTACATTGCTGCTGCAAGAGCTCTACGAAATGGTTCAACTAACATGGATGTTTTGGTTGCATTGGGGACATCAGCCTCCTATTTCTACTCTGTTTGTGCACTTCTATATGGTGCACTCACTGGGTTTTGGTCCCCAACTTACTTTGAAACAAGTGCAATGCTGATTACATTTGTGCTGTTAGGAAAATATCTAGAGTGTCTTGCAAAGGGGAAAACATCTGACGCCATCAAAAAGTTGGTAGAACTTGCACCAGCAACGGCAACATTGCTTATCAAGGATAAAGATGGAAGATATCTTGGAGAAAGGGAAATAGATGCTCTACTAATTCAGCCCGGTGACACATTGAAAGTTTCACCGGGTGCAAAATTACCTGCTGATGGTGTTGTTGCTTGGGGTTCAAGTTATGTAAATGAAAGTATGGTAACTGGTGAATCTGTACCCATTTCGAAGGAGGTCGGTTCATCAGTAATTGGAGGTACAATAAATTTACATGGTGCTCTACACGTACAAGCTACCAAAGTAGGTTCTGACACAGTTTTGAGCCAGATAATCAGTTTAGTTGAAACAGCGCAGATGTCCAAAGCTCCTATTCAGAAATTTGCTGATTTCATCGCTAGCATTTTTGTTCCTACAGTTGTTTTGTTGGCATTGTTGACGTTATTAGGATGGTACGCTGCTGGAGCTCTTCGTGCTTACCCAGAAAGTTGGCTTCCAGAAAATGGAAATTACTTTGTTTTCGCCCTTATGTTTTCTATATCAGTTGTGGTGATCGCGTGCCCCTGTGCTCTTGGCTTGGCAACACCAACTGCGGTCATGGTTGCAACCGGAGTCGGTGCTAACAATGGTGTGCTGATAAAAGGGGGCGATGCCTTAGAAAGGGCTCAAAAAATAAAGTATGTGATCTTTGATAAAACAGGTACCCTAACTCAGGGAAAGGCCTCGGTTACAACTACAAAAGTGTTTTCGGGAATGGATCGTGGAGAATTCCTCAAGTTGGTGGCTTCTGCTGAGGCTAGTAGTGAACATCCACTGGCAAAGGCAATAGTTGCATATGCCCGTCATTTTCATTTCTTTGATGATTCTGCTACCAAGGATGCCAAAAGCCATAGCAAAGACTCCGAAGTATCTGGTTGGCTTTTTGATGTAGCAGAATTTTCTACTCTTCCCGGCAGAGGAGTTCAATGCTTTATTAATGAAAAACAGATTTTGGTTGGTAATCGCAAGTTGATGACCGAAAGTGGAATCGACATCCCTGATGATGTAGAAAAATTTGTTGTAGAACTCGAAGAAAGTGCGAAAACAGGCATACTTGTCGCATATAACGGTAACTTAATTGGTGTTTTGGGGGTTGCAGACCCACTGAAAAGAGAGGCAGCTGTGGTAGTTGAGGGCCTGAGTAAAATGGGCATCCGACCGGTCATGGTTACTGGGGACAACTGGAGGACAGCGCAGGCTGTTGCTAAGGAGGTTGGCATTCACGATGTAAGGGCAGAGGTAATGCCTGCAGGAAAAGCCGATGTAGTTCGTTCGTTTCATAAGGATGGAATCACGGTTGCGATGGTGGGAGACGGAATCAATGATTCTCCAGCTCTAGCTGCAGCAGATGTTGGCATGGCAATTGGAGCAGGGACGGATATTGCCATTGAAGCGGCCAACTATGTTTTGATGAGGAACAACTTGGAGGATGTGATCACCGCCATTGATCTCTCAAGAAAGACTTTCTCTCGAATCAGATTAAATTATGTGTTTGCCATGGCTTACAATGTCATAGCAATCCCTGTTGCTGCCGGAGTTTTCTTTCCGTCGTTGGGGATTCAGTTGCCGCCATGGGCGGCTGGTGCATGCATGGCTTTGTCTTCTGTTAGTGTTGTATGTTCTTCTCTATTACTTAGGAGATACAGAAAACCAAGGCTGACCACTATACTAGAAATAACTGTAGAATAG

## >FcRAN1-Syrai_Xu

ATGGCGGCGAGCGTCCGACACCTCCAGCTCACCCAACTCTCCGCCGCCGGCGCCGGCGACGACGACGACTCCGGCGACCTCGAGGACGTGCGGCTCCTCGACGCGTACAAGAATTCGGAGGAGAACGATGAGGGAGTTGTCGGAGAGGCGACGATGAAGAGGATTCAGGTCGGCGTCACCGGCATGACCTGCGCGGCTTGCTCGAACTCCGTCGAAGCTGCTCTGATGAGCGTCAATGGCGTTCTCAGGGCTTCCGTCGCTCTGCTCCAGAACAAGGCCGACGTGGTCTTCGATCCCAGATTGGTCAAGGATGAAGATATCAAGAATGCAATCGAGGATGCTGGGTTTGAAGCTGAGATTCTACCTGAATCAAGTGCCATTGGAACAAAGCCTCAAGCGACCACGTTAGGGCAGTTCTCTATAGCTGGTATGACATGTGCGGCTTGTGTGAACTCGGTAGAAGGCATTTTACGAGATCTTCCCGGTGTCAAAAGGGCTGTCGTTGCCTTGGCTACTTCATTAGGTGAAGTTGAGTATGATCCAGCTGTTATCAGTAAAGAGGATATCGTCAATGCAATTGAAGACGCTGGTTTTGAAGGAGCCTTTGTACAGAGCAGTGAACAAGATAAGATTGTGTTGGGCGTTGCTGGCATATACAGTGAGATGGATGTACAGATTTTAGGAGGCATACTTAGCAACTTGAAAGGGGTGAGACAATTTCATTTTGACCGAATTTCAAGAGAACTTGAAGTTCTGTTTGATCCTGAAGTTATTCATTCAAGATCCTTAGTTGATGGGGTTGAAGGGGGAAGTAGCGGAAGGTTTAAATTACATGTTGCTAATCCTTATTCAAGAATGACTTCTAAAGATGTGGAAGAAGCCTCAAACATGTTTAGACTGTTCATCTCTAGTCTATTTCTCAGCGTTCCTGTCTTTCTCATACGAGTAGTTTGTCCACACATACCACTGATTTATTCCTTATTGCTTTGGCGATGTGGGCCCTTTCAAATGGGTGACTGGTTGAAGTGGGCGTTGGTGAGTGTCGTTCAATTTGTTGTTGGAAAGCGCTTCTACATTGCTGCTGCAAGAGCTCTACGAAATGGTTCAACTAACATGGATGTTTTGGTTGCATTGGGGACATCAGCCTCCTATTTCTACTCTGTTTGTGCACTTCTATATGGTGCACTCACTGGGTTTTGGTCCCCAACTTACTTTGAAACAAGTGCAATGCTGATTACATTTGTGCTGTTAGGAAAATATCTAGAGTGTCTTGCAAAGGGGAAAACATCTGACGCCATCAAAAAGTTGGTAGAACTTGCACCAGCAACGGCAACATTGCTTATCAAGGATAAAGATGGAAGATATCTTGGAGAAAGGGAAATAGATGCTCTACTAATTCAGCCCGGTGACACATTGAAAGTTTCACCGGGTGCAAAATTACCTGCTGATGGTGTTGTTGCTTGGGGTTCAAGTTATGTAAATGAAAGTATGGTAACTGGTGAATCTGTACCCATTTCGAAGGAGGTCGGTTCATCAGTAATTGGAGGTACAATAAATTTACATGGTGCTCTACACGTACAAGCTACCAAAGTAGGTTCTGACACAGTTTTGAGCCAGATAATCAGTTTAGTTGAAACAGCGCAGATGTCCAAAGCTCCTATTCAGAAATTTGCTGATTTCATCGCTAGCATTTTTGTTCCTACAGTTGTTTTGTTGGCATTGTTGACGTTATTAGGATGGTACGCTGCTGGAGCTCTTCGTGCTTACCCAGAAAGTTGGCTTCCAGAAAATGGAAATTACTTTGTTTTCGCCCTTATGTTTTCTATATCAGTTGTGGTGATTGCGTGCCCCTGTGCTCTTGGCTTGGCAACACCAACTGCGGTCATGGTTGCAACAGGAGTCGGTGCTAACAATGGTGTGCTGATAAAAGGGGGCGATGCCTTAGAAAGGGCTCAAAAAATAAAGTATGTGATCTTTGATAAAACAGGTACCCTAACTCAGGGAAAGGCCTCGGTTACAACTACAAAAGTGTTTTCGGGAATGGATCGTGGAGAATTCCTCAAGTTGGTGGCTTCTGCTGAGGCTAGTAGTGAACATCCACTGGCAAAGGCAATAGTTGCATATGCCCGTCATTTTCATTTCTTTAATGATTCTGCTACCAAGGATGCCGAAAGCCATAGCAAAGACTCCGAAGTATCTGGTTGGCTTTTTGATGTAGCAGAATTTTCTACTCTTCCCGGCAGAGGAGTTCAATGCTTTATTAATGAAAAACAGATTTTGGTTGGTAATCGCAAGTTGATGACCGAAAGTGGAATCGACATCCCTGATGATGTAGAAAAATTTGTTGTAGAACTCGAAGAAAGTGCGAAAACAGGCATACTTGTCGCATATAACGGTAACTTAATTGGTGTTTTGGGGGTTGCAGACCCACTGAAAAGAGAGGCAGCTGTGGTAGTTGAGGGCCTGAGTAAAATGGGCATCCGACCGGTCATGGTTACTGGGGACAACTGGAGGACAGCGCAGGCTGTTGCTAAGGAGGTTGGCATTCACGATGTAAGGGCAGAGGTAATGCCTGCAGGAAAAGCCGATGTAGTTCGTTCGTTTCATAAGGATGGAATCACAGTTGCGATGGTGGGAGACGGAATCAATGATTCTCCAGCTCTAGCTGCAGCAGATGTTGGCATGGCAATTGGAGCAGGGACGGATATTGCCATTGAAGCGGCCAACTATGTTTTGATGAGGAACAACTTGGAGGATGTGATCACCGCCATTGATCTCTCAAGAAAGACGTTCTCTCGAATCAGATTAAATTATGTGTTTGCCATGGCTTACAATGTCATAGCAATCCCTGTTGCTGCCGGAGTTTTCTTTCCGTCGTTGGGGATTCAGTTGCCGCCATGGGCGGCTGGTGCATGCATGGCTTTGTCTTCTGTTAGTGTTGTATGTTCTTCTCTATTACTTAGGAGATACAGAAAACCAAGGCTGACCACTATACTAGAAATAACTGTAGAATAG

## >FhRAN1(Fh.01G0000580)

ATGAAGAGGATTCAGGTCGGCGTCGCCGGCATGACCTGCGCGGCTTGCTCGAACTCCGTCGAAGCTGCTCTGATGAGCGTCAATGGCGTTCTCAGGGCTTCCGTCGCTTTGCTCCAGAACAAGGCCGACGTGGTCTTCGATCCCAGATTGGTCAAGGATGAAGATATCAAGAATGCAATCGAGGATGCTGGGTTTGAAGCTGAGATTCTACCTGAATCAAGTGCTGTTGGAACAAAGCCTCAAGCGACCATGTTAGGGCAGTTCTCTATAGGTGGCATGACATGTGCGGCTTGCGTGAACTCGGTAGAAGGCATTTTACGAGATCTTCCGGGTGTCAAAAGGGCTGGAGTTGCCTTGGCTACTTCATTGGGTGAAGTTGAGTATGATCCAGCTGTTATCAGTAAAGAGGATATCGTCAATGCAATTGAAGACGCTGGTTTTGAAGGAGCCTTTGTACAGAGCAGTGAACAAGATAAGATTGTGTTGGGCGTTGCTGGCATATACAGTGAGATGGATGTACAGATTTTAGGAGGCATACTTAGCAGCTTGAAAGGGGTGAGACAATTTCATTTTGACCCAATTTCAAGAGAACTTGAAGTTCTGTTCGATCCTGAAGTTATTCATTCAAGATCCTTAGTTGATGGGGTTGAAGGGGGAAGTAGCGGAAGGTTTAAATTACATGTTGCTAATCCTTATTCAAGAATGACTTCTAAAGATGTGGAAGAAGCCTCAAACATGTTTAGACTGTTCATCTCTAGTCTATTTCTCAGCGTTCCTGTCTTTCTCATACGAGTAGTTTGTCCACACATACCACTGATTTATTCCTTATTGCTTTGGCGATGTGGGCCCTTTCAAATGGGTGACTGGTTGAAGTGGGCGTTGGTGAGTGTCGTTCAATTTGTTGTTGGAAAGCGCTTCTACATTGCTGCTGCAAGAGCTCTACGAAATGGTTCAACTAACATGGATGTTTTGGTTGCATTGGGGACATCAGCCTCCTATTTCTACTCTGTTTGTGCACTTCTATATGGTGCACTCACTGGGTTTTGGTCCCCAACTTACTTTGAGACAAGTGCAATGCTGATTACATTTGTGCTGTTAGGAAAATATCTAGAGTGTCTTGCAAAGGGGAAAACATCTGATGCCATCAAAAAGTTGGTAGAACTTGCACCAGCAACAGCAACATTGCTTATCAAGTATAAAGATGGAAGATATCTTGGAGAAAGGGAAATAGATGCTCTACTAATTCAGCCCGGTGACACATTGAAAGTTTCACCGGGTGCAAAATTACCTGCTGATGGTGTTGTTGCTTGGGGTTCAAGTTATGTAAATGAAAGTATGGTAACTGGTGAATCTGTACCCATTTCGAAGGAGGTCAGTTCATCAGTAATTGGAGGTACAATAAATTTACATGGTGCTCTTCACGTACAAGCTACCAAAGTAGGTTCTGACACAGTTTTGAGCCAGATAATCAGTTTAGTTGAAACAGCGCAGATGTCCAAAGCTCCTATTCAGAAATTTGCTGATTTCATCGCTAGCATTTTTGTTCCTACAGTTGTTTTGTTGGCATTGTTGACGTTATTAGGATGGTACGCTGCTGGAGCTCTTCGTGCTTACCCAGAAAGTTGGCTTCCAGAAAATGGAAATTACTTTGTTTTCGCCCTTATGTTTTCTATATCAGTTGTGGTGATTGCATGCCCCTGTGCTCTTGGCTTGGCAACACCAACTGCGGTCATGGTTGCAACAGGAGTCGGTGCTAACAATGGTGTGCTGATAAAAGGGGGCGATGCCTTAGAAAGGGCTCAAAAAATAAAGTATGTGATCTTTGATAAAACAGGTACCCTAACTCAGGGGAAGGCCTCGGTTACAACTACAAAAGTGTTTTCGGGAATGGATTGTGGAGAATTCCTCAAGTTGGTGGCTTCTGCAGAGGCTAGTAGTGAACATCCACTGGCGAAGGCAATAGTTGCATATGCCCATCATTTTCATTTCTTTGATGATTCTGCTACCAAGGATGCTGAAAGCCATAGCAAAGACTCTGAAGTATCTGGTTGGCTTTTTGATGTAGCAGAATTTTCTGCTCTTCCAGGCAGAGGAGTTCAATGCTTTATTAATGAAAAACAGATTTTGGTTGGTAATCGCAAGTTGATGTCCGAAAGTGGAATCGACATCCCTGATGATGTAGAAAAATTTGTTGTAGAACTCGAAGAAAGTGCGAAAACAGGCATACTTGTCACATATAACGGTAACTTAATTGGTGTTTTGGGGGTTGCAGATCCACTGAAAAGAGAGGCAGCTGTGGTAGTTGAGGGCCTGAGTAAAATGGGCATCCGACCGGTCATGGTTACCGGGGACAACTGGAGGACAGCGCAGGCTGTTGCTAAGGAGGTTGGCATTCATGATGTAAGGGCAGAGGTAATGCCTGCAGGAAAAGCCGATGTAGTTCGTTCGTTTCAAAAGGATGGAAGCACGGTTGCGATGGTGGGAGACGGAATCAATGATTCTCCAGCTCTAGCTGCAGCAGATGTTGGCATGGCAATTGGAGCAGGGACGGATATTGCCATTGAAGCGGCTGACTATGTTTTAATGAGGAACAACTTGGAGGATGTGATCACCGCCATTGATCTCTCAAGAAAGACGTTCTCTCGAATCAGATTAAATTATGTGTTTGCCATGGCTTACAATGTCATAGCAATCCCTGTTGCTGCCGGAGTTTTCTTTCCATCGTTGGGGATTCAGTTGCCACCATGGGCGGCTGGTGCGTGCATGGCTTTGTCTTCTGTTAGTGTTGTATGTTCTTCTTTATTACTTAGGAGATACAGAAAACCGAGGCTGACCACTATACTAGAAATAACTGTAGAATAG

## >FmRAN1(Fm.01G0001630)

ATGTTAGGGCAGTTCTCTATAGGTGGCATGACATGTGCGGCTTGCGTGAATACGGTAGAAGGCATTTTACGAGATCTTCCCGGTGTCAAAAGGGCTGTAGTTGCCTTGGCTACTTCATTAGGTGAAGTTGAGTATGATCCAGCTGTTATCAGTAAAGAGGATATCGTCAATGCAATTGAAGACACTGGTTTTGAAGGAGCCTTTCTACAGAGCAGTGAACAAGATAAGATTGTTTTGGGCGTTGCTGGCATATACAGTGAGATGGACGTACAGATTTTAGGAGGCATACTTAGCAACTTGAAAGGGGTGAGACAATTTCATTTTGACCGAATTTCAAGAGAACTTGAAGTTCTATTCGATCCTGAAGTTATTCATTCAAGATCCTTAGTTGATGGGGTTGAAGGGGGAAGTAGCGGAAGGTTTAAATTACATGTTGCTAATCCTTATTCAAGAATGACTTCTAAAGATGTGGAAGAAGCCTCAAACATGTTTAGACTGTTCATCTCTAGTCTATTTCTCAGCGTTCCTGTCTTTCTCATACGAGTAGTTTGTCCACACATACCGCTGATTTATTCCTTATTGCTTTGGCGATGTGGGCCCTTCCAAATGGGTGATTGGTTGAAGTGGGCGTTGGTGAGTGTCGTTCAATTTGTTGTTGGAAAGCGCTTCTACATTGCTGCTGCAAGAGCTCTACGAAATGGTTCAACTAACATGGATGTTTTGGTTGCATTGGGGACATCGGCCTCCTATTTCTACTCTGTTTGTGCACTTCTATATGGTGCATTCACTGGGTTTTGGTCCCCAACTTACTTTGAAACAAGTGCAATGCTGATTACATTTGTGCTATTAGGAAAATATCTAGAGTGTCTTGCAAAGGGGAAAACATCTGACGCCATCAAAAAGTTGGTAGAACTTGCACCAGCAACGGCAACATTGCTTATCAAGGATAAAGATGGAAGATGTCTTAGAGAAAGGGAAATAGATGCTCTACTAATTCAGCCTGGTGACACATTGAAAGTTTCACCTGGTGCGAAAGTACCTGCTGATGGTGTTGTTGCTTGGGGTTCAAGTTATGTAAATGAAAGTATGGTAACTGGTGAATCTGTACCCATTTCAAAGGAGGTTAGTTCATCAGTAATTGGAGGTACAATAAATTTACATGGTGCTCTACACGTACAAGCTACCAAAGTAGGTTCTGACACAGTTTTGAGCCAGATAATCAGTTTAGTTGAAACAGCGCAGATGTCCAAAGCTCCTATTCAGAAATTTGCTGATTTTATCGCTAGCATTTTTGTTCCTACAGTTGTTATATTGGCATTGTTGACGTTAATGGGATGGTACGCTGCTGGAGCTCTTGGTGCTTACCCAGAAAGTTGGCTTCCAGAAAATGGAAATTACTTTGTTTTCGCCCTTATGTTTTCTATATCAGTTGTGGTGATCGCGTGCCCCTGTGCTCTCGGCTTGGCAACACCAACTGCGGTCATGGTTGCAACAGGAGTTGGTGCTAACAATGGTGTGCTGATAAAAGGAGGCGATGCCTTAGAAAGGGCTCAAAAAATAAAGTATGTGATCTTTGATAAAACAGGTACCCTAACTCAGGGAAGGGCCTCGGTTACAACTACAAAAGTGTTTTCGGGAATGGATCGTGGAGAATTCCTCAAGTTGGTGGCTTCTGCAGAGGCTAGTAGTGAACATCCACTGGCGAAGGCAATAGTTGCATATGCCCGTCATTTTCATTTCTTTGATGATTCTGCTACCAAGGATGCCGAAAGCCATAGCAAAGACTCTGAAGTATCTGGTTGGCTTTTTGATGTAACAGAATTTTCTACTCTTCCCGGCAGAGGAGTTCAATGCTTTATTAATGAAAAACAGATTTTGGTTGGTAATCGCAACTTGATGACCGAAAGTGGAATTGACATCCCTGACGATGTAGAAAAATTTGTTGTGGAACTCGAAGAAAGTGCGAAAACAGGCATACTCGTCTCATATAACGGTAACTTAATTGGTGTTTTGGGGGTTGCAGACCCACTGAAAAGAGAGGCAGCTGTGGTAGTTGAGGGCCTGAGTAAAATGGGCGTCCGACCGGTCATGGTTACCGGGGACAACTGGAGGACAGCACAGGCTGTTGCTAAGGAGGTTGGCATTCACGATGTGAGGGCAGAGGTAATGCCTGCAGGAAAAGCCGATGTAGTTCGTTCATTTCAAAAGGATGGAAGCACGGTTGCGATGGTGGGAGACGGAATCAATGATTCTCCAGCTCTAGCTGCAGCAGATGTTGGCATGGCAATTGGAGCAGGGACAGATATTGCCATTGAAGCGGCCGACTATGTTTTGATGAGGAACAACTTGGAGGATGTGATCACCGCCATTGATCTCTCAAGAAAGACGTTCTTCCGAATCAGATTAAATTATGTGTTTGCCATGGCTTACAATGTCATAGCAATCCCTGTTGCTGCCGGAGTTTTCTTTCCGTCGTTGGGGATTCAGTTGCCGCCATGGGCGGCTGGTGCTTGCATGGCTCTGTCTTCTGTTAGTGTTGTATGTTCTTCTTTATTACTTAGGAGATACAGAAAACCAAGGCTGACCACTATACTAGAAATAACTGTAGAATAG

## >MaRAN1(XM_010089630.2)

CTTTTCCCTTCCCTTTCTCTCTCTCTCTCTCTTTAAATCTCAGCCCAAAATTTTCCTCTCTTTTCTCTTCACACAAACAAATAAAACCTCTGTTTGCCCCTCCATTTTTCTTACCCAAATATGGCGCCGAACAGCAGAAACCTCCAGCTCACCCAGCTCTCCGTCTCCGGCGCCGGCGACGGCGACCTCGAGGAGGTGCGACTCCTCGACGCGTACGAGAATTTGGAGGAGGAGGGAGTAATCGGAGAGGGGACGGTGAAGAGGATTCAGGTCGGCGTCACCGGAATGACCTGCGCTGCTTGCTCGAATTCCGTTGAAGCAGCTCTGATGAGCGTTCATGGCGTTCTCAGGGCTTCCGTTGCTCTTCTTCAGAACAAGGCTGACGTGGTCTTTGATCCCAGATTGGTCAAGGATGAAGACATCAAGAGCGCAATCGAGGATGCTGGGTTTGAAGCAGAGATTCTACCTGAATCCAGTGCAGTTGGGACAAAGCCTCAAGGAACCCTGTCAGGGCAGTTCTCAATAGGAGGCATGACATGTGCAGCCTGCGTGAACTCCGTTGAGGGCATCTTGAGAGATCTTCCTGGTGTTGAAAGGGCGGTAGTTGCATTGGCAACTTCATTAGGTGAAGTTGAGTATGATCCAGCCATAATCAGTAAAGAGGATATAGTCAATGCGATTGAAGATGCTGGTTTTGAAGGAGCCTTTGTACAGAGCAGTGAACAAGACAAGATTGTCTTGGGGGTTACTGGCATATACAGTGATGTGGATGTACAGCTTTTAGGGGGCATAATTAGCAACTTAAAGGGGGTGAGGCAATTTTATTTTGATCGAATTACAAGAGAACTTGAAGTCCTATTTGATCCTGAAGTTGTCAATTCAAGATCATTAGTTGATGGGATTGAAGGGGGAAGTAGCGGAAGGTTTAAATTACGTGTCGCAAACCCTTATTCAAGAATGACTTCTAAGGATGTGGAAGAAGCCTCAAATATGTTTAGATTGTTCATCTCTAGTCTATTTCTCAGTGTTCCTGTCTTTCTAATACGGGTAGTTTGTCCGCACATACCACTGATTTATTCCTTATTGCTCTGGCGATGTGGGCCCTTCCAAATGGGTGATTGGTTGAAGTGGGCATTGGTGAGTGTTGTTCAATTTGTTGTTGGAAAACGCTTCTACATTGCTGCTGCCAGAGCTCTGCGAAATGGTTCCACTAATATGGATGTTTTGGTTGCACTGGGAACATCGGCCTCTTATTTCTACTCTGTTTGTGCCCTTCTGTATGGTGCAGTCACTGGGTTTTGGTCGCCAACGTACTTTGAAACAAGTGCAATGCTGATCACGTTTGTGCTCTTAGGAAAGTATCTGGAGTGTCTTGCAAAGGGGAAAACATCTGACGCTATCAAGAAGTTGGTAGAACTTGCACCAGCAACAGCAATGTTGCTTATCAAAGATACAGACGGAAGATGTATCAGAGAAAGGGAAATAGATGCTCTATTAATTCAGCCTGGTGACACATTGAAAGTTTTACCGGGTGCAAAGGTTCCTGCTGATGGTCTTGTTGCTTGGGGTACAAGTTATGTGAATGAAAGTATGGTAACTGGTGAATCTGTACCTGTTTCAAAGCAGGTCGGTTCAACAGTTATTGGAGGTACAATAAATTTACATGGCGCTTTACACATACAAGCTACCAAAGTAGGTTCTGATACAGTTTTGAGTCAGATTATCAGTTTAGTTGAGATGGCACAGATGTCCAAAGCTCCCATACAGAAATTTGCAGATTTTATTGCAAGCATTTTTGTTCCTACTGTTGTTATGTTGGCGTTGTTGACATTATTGGGATGGTACATGGCTGGAGCTCTTGGAGCCTATCCAGAAAGTTGGCTCCCAGAAAATGGAAATCACTTCGTCTTCGCCCTTATGTTTTCTATATCAGTTGTGGTGATTGCATGCCCTTGTGCTCTTGGCTTGGCAACACCAACTGCTGTCATGGTTGCAACAGGAGTTGGTGCTAACAATGGTGTGCTGATAAAAGGAGGGGATGCCTTGGAAAGGGCTCAAAAAATTAAGTATGTGATATTTGATAAAACAGGTACCCTAACTCAGGGAAAGGCTTCGGTTACAACCACAAAAGTTTTCACGGGAATGGACCGTGGAGAATTCCTTAAATTGGTGGCTTCTGCAGAGGCTAGTAGCGAACATCCACTGGCAAAGGCAATAGTTGCATATGCCCAGCATTTTCATTTCTTTGATGATTCTGCTACTAAGGATGCCGAAAGCAATAACAAAGACTCTGCAGTATCTGGTTGGCTTTTTGATGTTGCAGAATTTTCTGCTTTGCCTGGAAGAGGAGTCCAATGCTTTATTGACGGAAAACAGGTTTTGGTTGGTAATCGCAAGTTGATGACTGAGAGTGGAATCAACATCCTTGATAATGTGGAAAAATTTGTGGTAGATCTTGAAGACAGTGCGAAAACAGGCATACTTGTCGCATATGATGGTAATTTAATCGGTGTTTTGGGGGTTGCAGACCCACTGAAAAGGGAAGCTGCTGTGGTAGTTGAGGGCCTGAGAAAAATGGGCGTTCGACCAGTCATGGTTACTGGGGACAATTGGAGGACAGCACGGGCCGTTGCTAAGGAGGTTGGCATTCACGATGTAAGGGCAGAGGTAATGCCTGCAGGAAAAGCTGATGTAATTCGTTCGTTTCAAAATGATGGAAGTACGGTCGCAATGGTTGGCGATGGAATCAATGATTCTCCGGCTCTAGCCGCAGCAGATGTTGGTATGGCAATTGGAGCAGGGACAGATATTGCCATTGAAGCGGCTGATTATGTTTTGATGAGGAGCAATTTGGAGGATGTGATTACCGCCATTGATCTCTCGAGAAAGACGTTCTCTCGAATCAGATTGAATTATGTGTTTGCCATGGCTTACAATGTCGTAGCGATCCCTATTGCTGCCGGAGTTTTCTTTCCGTCGTCGGGGATTCAGTTGCCTCCATGGGCAGCTGGCGCGTGCATGGCTCTGTCTTCTGTTAGTGTTGTATGTTCTTCTTTATTACTTAGGAGATACAGAAAACCAAGGCTTACCACTATACTAGAAATAACTGTAGAATAGAAAGTGTGTCAAGGAAAAAAGTTAAAAATAAAAAGTTAAAATTTCACATTCATTTCACAGGAAGGTTACTGATATTTACATAAAGTGGGTCTCGATGGAATTTGGGCCCAAATTTTGTGTTTTTTTTTTGTTTTTTTTGTTTTTTTGTTTTTCCCCTTTTTTTCCCACGTGGTGTAATTCTACCACCCCAGTCCTTGTAAATGCTACTTGTATAATAATTTTCTTTGGTTTTAACCTTTTGTCAATCCA

## >FmAG

ATGGCGTTCCAAAACAAGGAGATGATGTCAATGGAAACGGATTCTCCTACTCAAAGGAAAATGGGAAGGGGAAAGATCGAGATTAAGAGGATCGAAAACACAACGAATCGGCAAGTGACATTTTGCAAACGTAGAAACGGATTGTTGAAGAAGGCCTATGAACTCTCTGTTCTCTGTGATGCTGAGGTTGCCCTCATCGTCTTCTCCACCCGTGGACGCCTCTATGAGTATGCAAACCAGAGCGTCAAATCAACGATTGACAGGTACAAGAAAGCATGCTCTGATTCTTCAAATACCGGATCTGTTGCCGAAGCTAATGCTCAGTTCTACCAGCAAGAATCCGCAAAACTCCGGAACCAAATCGAGGCTACGCAGAAGGGAATTAGGGACATGCTGGGTGAGAATCTGAGCAGCTCAAGTGCCAGAGAACTCAAAAACCTGGAGGGTAAATTGGAGAAAGCACTTAGCAGAATCAGAACCAAAAAGAATGAGCTGTTGTTCCAAGAAATCGAGTACATGCAGAAAAGGGAAGTTGACTTGCACAACAATAACCAGCTTCTCCGAGCAAAGATAGCTGAAAATGAGAGATGCCAGCAGAACATGAACGTGATGGCAGGAGGAGGAATGTACGAGCTCATGCCGCAGTCTCAGGCGCAGACACCGGCATTCGACACTCGGAATTATTTTCAAGTGCATGCCCTACAGCCGGCCAATCATCAGTACTCTCACCAAGATTCCATGGCCCTCCAATTAGTGTAA

## >FhAG1

ATGTCGTACCAAAACAAGGAGATGATGTCAATGGAAATGGATTCAACTACTCAAAGGAAAATGGGAAGGGGAAAGATCGAGATTAAGAGGATCGAAAACACGACGAATCGGCAAGTGACATTTTGCAAACGTAGAAACGGATTGTTGAAGAAGGCCTATGAACTCTCTGTTCTCTGTGATGCTGAGGTTGCCCTCATCGTCTTCTCCACCCGTGGACGCCTCTATGAGTATGCAAACCAGAGCGTCAAATCAACGATTGACAGGTACAAGAAAGCATGCTCTGATTCTTCAAATACTGGATCTGTTGCCGTAGCTAATGCTCAGTTCTACCAGCAAGAATCCGCAAAACTCCGGAACCAAATCGAGGCTACGCAGAAGGGAATTAGGGACATACTGGGCGAGAGTCTGAGCAGCTCAAGTGCCAGAGAACTCAAAAACCTGGAGGGTAAATTGGAGAAAGCAATTAGCAAAATCAGAACCAAAAAGAATGAGCTGTTGTTCCAAGAAATCGAGTACATGCAGAAAAGGGAAGTTGACTTGAACAACAATAACCAGCTTCTCCGAGCAAAGATAGCTGAAAATGAGAGAAGCCAGCAGAACATGAACGTGATGGCAGGAGGAGGAAGCTATGAGCTCATGCCGCAGTCTCAGGCGCATCAGGAGCATACGCAGGCATTCGACTCTCGGAATTATTTTCAAGTGAATGCTCTACAGCCGGCCAATCATCAGTACTCTCGACAAGATCCCATGGCCCTCCAATTAGTCTAA

## >FhAG2

ATGTCGTACCAAAACAAGGAGATGATGTCAATGGAAATGGATTCTCCTACTCAAAGGAAAATGGGAAGGGGAAAGATCGAGATTAAGAGGATCGAAAACACGACGAATCGGCAAGTGACATTTTGCAAACGTAGAAACGGATTGTTGAAGAAGGCCTATGAACTCTCTGTTCTCTGTGATGCTGAGGTTGCCCTCATCGTCTTCTCCAGCCGTGGACGCCTCTATGAGTATGCAAACCAGAGCGTCAAATCAACGATTGACAGGTACAAGAAAGCATGCTCTGATTCTTCAAATACTGGATCTGTTGCCGAAGCTAATGCTCAGTTCTACCAGCAAGAATCTGCAAAACTCCGGAACCAAATCGAGGCTATGCAGAAGGGAATTAGGGACATGCTGGGTGAGAGTCTGAGCAGCTCAAGTGCCAGAGAACTCAAAAACCTAGAGGGTAAACTGGAGAAAGCAATCAGCAAAATCAGAACCAAAAAGAATGAGCTGTTGTTCCAAGAAATCGAGTACATGCAGAAAAGGGAAGTCGACTTGCACAACAGTAACCAGCTTCTCCTAGCAAAGATAGCTGAAAATGAGAGAAGCCAGCCGAATATGAACGTGATGGCAGGAGAAGGAATCTACGAGCTCATACCGCAGTCTCAGGCGCAGACACAGGCCTTCGACACTTGGAATTATTTTCAAGTGAATGCCCTACAGCCGGCCAATCATCAGTACTCTCACCAAGATCCCATGGCCCTCCAATTACTAAATATGGCTGGCTGGTAG

## >FhAG3

ATGTCGTACCAAAACAAGGTGAAGATGTCAATGGAAATGGATTCTCCTACTCAAAGGAAAATGGGAAGGGGAAAGATCGAGATTAAGAGGATCGAAAACACGACGAATCGGCAAGTGACATTTTGCAAACGTAGAAACGGATTGTTGAAGAAGGCCTATGAACTCTCTGTTCTCTGTGATGCTGAGGTTGCCCTCATCGTCTTCTCCAGCCGTGGACGCCTCTATGAGTATGCAAACCAGAGCGTTAAATCAACGATTGACAGGTACAAGAAAGCATGCTCTGATTCTTCAAATACTGGATCTGTTGCCGAAGCTAATGCTCAGTTCTACCAGCAAGAATCCGCAAAACTCCGGAACCAAATCGAGGCTACGCAGAAGGGAATTAGGGATATGCTGGGTGAGAGTCTGAGCAGCTCAAGTGCCAGAGAACTAAAAAACCTGGAGGGTAAATTGGAGAAAGCAATAAGCAAAATCAGAACCAAAAAGAATGAGCTGTTGTTCCAAGAAATTGAGTACATGCAGAAAAGGGAAGTCGACTTGCACAACGATAACCAGCTTCTCCTAGCAAAGATAGCTGAAAATGAGAGAAGCCAGCAGAACATGAACGTGATGGCAGGAGGAGGAAGCTACGAGCTCATACCGCAGTCTCAGGCGCAGACACATGCATTCGACACTTGGAATTATTTTCAAGTGAATGCCCTACAGCCGGCCAATCATCAGTACTCTCACCAAGATCCCATGGCCCTCCAATTAGTCTAA

## >FcAG-Dottato

ATGGCGTACCAAAACAAGGAGATGATGTCAATGGAAATGGATTCTCTTACTCAAAGGAAAATGGGAAGGGGAAAGATCGAGATTAAGAGGATCGAAAACACAACGAATCGGCAAGTGACATTTTGCAAACGTAGAAACGGATTGTTGAAGAAGGCCTATGAACTCTCTGTTCTCTGTGATGCTGAGGTTGCCCTCATCGTCTTCTCCAGCCGTGGACGCCTCTATGAGTATGCAAACCAGAGCCAAAGCCCTTCCATATCTCCTCGACACTTCCTTAATAGCACGTCAGTTTTAGACATCAAGAAAGCAAAGGCGTCAAATCAACGATTGACAGGTACAAGAAAAGCATGCTCTGATTCTTCAAGTACTGGATCTGTTGCCGTAGCTAATGCTCAGTTCTACCAGCAAGAATCCGCAAAACTCCGGAACCAAATCGAGGCTACGCAGAAGGGAATTAGGGACATGCTGGGTGAGAGTCTGAGCAGCTCAAGTGCCAGAGAACTCAAAAACCTGGAGGGTAAATTGGAGAAAGCAATTAGCAAAATCAGAACCAAAAAGAATGAGCTGTTGTTCCAAGAAATCGAGTACATGCAGAAAAGGGAAGTTGACTTGAACAACAATAACCAGCTTCTCCGAGCAAAGATAGCTGAAAACGAGAGAAGCCAGCAGAACATGAACGTGATGGACGAGCTCATGCCGCAGTCTCAGGCGCAGACACAGGCATTCGACACTCGGAATTATTTTCAAGTGAATGCCCAACAGCCGGCCAATCATCAGTACTCTCACCAAGATCCCATGGCCCTCCAATTAGTGTAA

## >FcAG-Dottato*

ATGTCGTACCAAAACAAGGTGAAGATGTCAATGGAAATGGATTCTTCTACTCAAAGGAAAATGGGAAGGGGAAAGATCGAGATTAAGAGGATCGAAAACACGACGAATCGGCAAGTGACATTTTGCAAACGTAGAAACGGATTGTTGAAGAAGGCCTATGAACTCTCTGTTCTCTGTGATGCTGAGGTTGCCCTCATCGTCTTCTCCAGCCGTGGACGCCTCTATGAGTATGCAAACCAGAGCGTCAAATCAACGATTGACAGGTACAAGAAAACATGCTCTGATTCTTCAAGTACTGGATCTGTTGCCGTAGCTAATGCTCAGTTCTACCAGCAAGAATCCGCAAAACTCCGGAACCAAATCGAGGCTACGCAGAAGGGAATTAGGGACATGCTGGGTGAGAGTCTGAGCAGCTCAAGTGCCAGAGAACTCAAAAACCTGGAGGGTAAATTGGAGAAAGCAATTAGCAAAATCAGAACCAAAAAGAATGAGCTGTTGTTCCAAGAAATCGAGTACATGCAGAAAAGGGAAGTTGACTTGAACAACAATAACCAGCTTCTCCGAGCAAAGATAGCTGAAAACGAGAGAAGCCAGCAGAACATGCACGTGATGGACGAGCTCATGCCGCAGTCTCAGGCGCAGACACAGGCATTCGACACTCGGAATTATTTTCAAGTGAATGCCCAACAGCCGGCCAACCATCAGTACTCTCACCAAGATCCCATGGCCCTCCAATTAGTGTAA

## >FcAG-Pistil

ATGGCGTTCCAAAACAAGGAGATGATGTCAATGGAAATGGATTCTTCTACTCAAAGGAAAATGGGAAGGGGAAAGATCGAGATTAAGAGGATCGAAAACACGACGAATCGGCAAGTGACATTTTGCAAACGTAGAAACGGATTGTTGAAGAAGGCCTATGAACTCTCTGTTCTCTGTGATGCTGAGGTTGCCCTCATCGTCTTCTCCAGCCGTGGACGCCTCTATGAGTATGCAAACCAGAGCGTCAAATCAACGATTGACAGGTACAAGAAAACATGCTCTGATTCTTCAAGTACTGGATCTGTTGCCGTAGCTAATGCTCAGTTCTACCAGCAAGAATCCGCAAAACTCCGGAACCAAATCGAGGCTACGCAGAAGGGAATTAGGGACATGCTGGGTGAGAGTCTGAGCAGCTCAAGTGCCAGAGAACTCAAAAACCTGGAGGGTAAATTGGAGAAAGCAATTAGCAAAATCAGAACCAAAAAGAATGAGCTGTTGTTCCAAGAAATCGAGTACATGCAGAAAAGGGAAGTTGACTTGAACAACAATAACCAGCTTCTCCGAGCAAAGATAGCTGAAAACGAGAGAAGCCAGCAGAACATGAACGTGATGGACGAGCTCATGCCGCAGTCTCAGGCGCAGACACAGGCATTCGACACTCGGAATTATTTTCAAGTGAATGCCCTACAGCCGGCCAATCATCAGTACTCTCACCAAGATCCCATGGCCCTCCAATTAGTGTAA

## >FcAG-Purple peel

ATGGCGTACCAAAACAAGGAGATGATGTCAATGGAAATGGATTCTTCTACTCAAAGGAAAATGGGAAGGGGAAAGATCGAGATTAAGAGGATCGAAAACACGACGAATCGGCAAGTGACATTTTGCAAACGTAGAAACGGATTGTTGAAGAAGGCCTATGAACTCTCTGTTCTCTGTGATGCTGAGGTTGCCCTCATCGTCTTCTCCAGCCGTGGACGCCTCTATGAGTATGCAAACCAGAGCGTCAAATCAACGATTGACAGGTACAAGAAAACATGCTCTGATTCTTCAAGTACTGGATCTGTTGCCGTAGCTAATGCTCAGTTCTACCAGCAAGAATCCGCAAAACTCCGGAACCAAATCGAGGCTACGCAGAAGGGAATTAGGGACATGCTGGGTGAGAGTCTGAGCAGCTCAAGTGCCAGAGAACTCAAAAACCTGGAGGGTAAATTGGAGAAAGCAATTAGCAAAATCAGAACCAAAAAGAATGAGCTGTTGTTCCAAGAAATCGAGTACATGCAGAAAAGGGAAGTTGACTTGAACAACAATAACCAGCTTCTCCGAGCAAAGATAGCTGAAAACGAGAGAAGCCAGCAGAACATGAACGTGATGGACGAGCTCATGCCGCAGTCTCAGGCGCAGACACAGGCATTCGACACTCGGAATTATTTTCAAGTGAATGCCCTACAGCCGGCCAATCATCAGTACTCTCACCAAGATCCCATGGCCCTCCAATTAGTCTAA

## >FcAG-Gall

ATGGCGTACCAAAACAAGGTGAAGATGTCAATGGAAATGGATTCTTCTACTCACAGGAAAATGGGAAGGGGAAAGATCGAGATTAAGAGGATCGAAAACACGACGAATCGGCAAGTGACATTTTGCAAACGTAGAAACGGATTGTTGAAGAAGGCCTATGAACTCTCTGTTCTCTGTGATGCTGAGGTTGCCCTCATCGTCTTCTCCAGCCGTGGACGCCTCTATGAGTATGCAAACCAGAGCGTCAAATCAACGATTGACAGGTACAAGAAAACATGCTCTGATTCTTCAAGTACTGGATCTGTTGCCGTAGCTAATGCTCAGTTCTACCAGCAAGAATCCGCAAAACTCCGGAACCAAATCGAGGCTACGCAGAAGGGAATTAGGGACATGCTGGGTGAGAGTCTGAGCAGCTCAAGTGCCAGAGAACTCAAAAACCTGGAGGGTAAATTGGAGAAAGCAATTAGCAAAATCAGAACCAAAAAGAATGAGCTGTTGTTCCAAGAAATCGAGTACATGCAGAAAAGGGAAGTTGACTTGAACAACAATAACCAGCTTCTCCGAGCAAAGATAGCTGAAAACGAGAGAAGCCAGCAGAACATGAACGTGATGGACGAGCTCATGCCGCAGTCTCAGGCGCAGACACAGGCATTCGACACTCGGAATTATTTTCAAGTGAATGCCCTACAGCCGGCCAATCATCAGTACTCTCACCAAGATCCCATGGCCCTCCAATTAGTGTAA

## >FcAG2-Gall

ATGGCGTACCAAAACAAGGAGAAGATGTCAGTAATAATGGATTCTCCTACTCAAAGGAAAATGGGAAGGGGAAAGATCGAGATTAAGAGGATCGAAAACACGACGAATCGGCAAGTGACATTTTGCAAACGTAGAAACGGATTGTTGAAGAAGGCCTATGAACTCTCTGTTCTCTGTGATGCTGAGGTTGCCCTCATCGTCTTCTCAAGCCGTGGACGCCTCTATGAGTATGCAAACCAGAGCGTCAAATCAACGATTGACAGGTACAAGAAAGCATGCTCTGATTCTTCAAATACTGGATCTGTTGCCGAAGCTAATGCTCAGTTCTACCAGCAAGAATCCGCAAAACTCCAAAACCAAATCGAGGCTACGCAGAAGGGAATTAGGGACATGCTGGGTGAGAGTCTGAGCAGCTCAAGTGCCAGAGAACTCAAAAACCTGGAGGGTAAATTGGAGAAAGCAATCAGCAAAATCAGAACCAAAAAGAATGAGCTGTTGTTCCAAGAAATCGAGTACATGCAGAAAAGGGAAGTCGACTTGCACAACAATAACCAGCTTCTCCGAGCAAAGATAGCTGAAAATGAGAGAAGCCAGCAGAACATGAACGTGATGGCAGGAGGAGGAAGCTACGAGCTCATGCCGCAGTCTCAGGCGCAGACGCAGGCATTCGACCCTTGGAATTATTTTCAAGTGAATGCCCTACAGCCGGCCAATCATCAGTACTCTCACCAAGATCCCATGGCCCTCCAATTAGTGTAA

## >FcAG3-Gall

ATGGCGTTCCAAAACAAGGAGAAGATGTCAATGGAAATGGATTCTTCTACTCAAAGGAAAATGGGAAGGGGAAAGATCGAGATTAAGAGGATCGAAAACACGACGAATCGGCAAGTGACATTTTGCAAACGTAGAAACGGATTGTTGAAGAAGGCCTATGAACTCTCTGTTCTCTGTGATGCTGAGGTTGCCCTCATCGTCTTCTCCAGCCGTGGACGCCTCTATGAGTATGCAAACCAGAGCGTCAAATCAACGATTGACAGGTACAAGAAAGCATGCTCTGATTCTTCAAATACTGGATCTGTTGCCGAAGCTAATGCTCAGTTCTACCAGCAAGAATCCGCCAAACTCCGGAACCAAATCGAGGCTACGCAGAAGGGAATTAGGGACATGCTGGGTGAGAGTCTGAGCAGCTCAAGTGCCAGAGAACTCAAAAACCTAGAAGGTAAATTGGAGAAAGCAATCAGCAAAATCAGAACCAAAAAGAATGAGCTGTTGTTCCAAGAAATTGAGTACATGCAGAAAAGGCAAGTCGACTTGCACAACAATAACCAGCTTCTCCGAGCAAAGATAGCTGAAAATGAGAGAAGCCAGCAGAACATGAACGTGATGGCAGGAGGAGGAAGCTACGAGCTCATGCCGCAGTCTCAGGCGCAGACACAGGCATTCGACCCTTGGAATTATTTTCAAGTGAATGCCCTACAGCCGGCCAATCATCAGTACTCTCACCAAGATCCCATGGCCCTCCAATTAGTGTAA

## >FcAG2-Stamen

ATGGCGTACCAAAACAAGGAGAAGATGTCAGTAATAATGGATTCTCCTACTCAAAGGAAAATGGGAAGGGGAAAGATCGAGATTAAGAGGATCGAAAACACGACGAATCGGCAAGTGACATTTTGCAAACGTAGAAACGGATTGTTGAAGAAGGCCTATGAACTCTCTGTTCTCTGTGATGCTGAGGTTGCCCTCATCGTCTTCTCAAGCCGTGGACGCCTCTATGAGTATGCAAACCAGAGCGTCAAATCAACGATTGACAGGTACAAGAAAGCATGCTCTGATTCTTCAAATACTGGATCTGTTGCCGAAGCTAATGCTCAGTTCTACCAGCAAGAATCCGCAAAACTCCAAAACCAAATCGAGGCTACGCAGAAGGGAATTAGGGACATGCTGGGTGAGAGTCTGAGCAGCTCAAGTGCCAGAGAACTCAAAAACCTGGAGGGTAAATTGGAGAAAGCAATCAGCAAAATCAGAACCAAAAAGAATGAGCTGTTGTTCCAAGAAATCGAGTACATGCAGAAAAGGGAAGTCGACTTGCACAACAATAACCAGCTTCTCCGAGCAAAGATAGCTGAAAATGAGAGAAGCCAGCAGAACATGAACGTGATGGCAGGAGGAGGAAGCTACGAGCTCATGCCGCAGTCTCAGGCGCAGACGCAGGCATTCGACCCTTGGAATTATTTTCAAGTGAATGCCCTACAGCCGGCCAATCATCAGTACTCTCACCAAGATCCCATGGCCCTCCAATTAGTGTAA

## >FcAG3-Stamen

ATGGCGTACCAAAACAAGGAGAAGATGTCAGTAATAATGGATTCTCCTACTCAAAGGAAAATGGGAAGGGGAAAGATCGAGATTAAGAGGATCGAAAACACGACGAATCGGCAAGTGACATTTTGCAAACGTAGAAACGGATTGTTGAAGAAGGCCTATGAACTCTCTGTTCTCTGTGATGCTGAGGTTGCCCTCATCGTCTTCTCCAGCCGTGGACGCCTCTATGAGTATGCAAACCAGAGCGTCAAATCAACGATTGACAGGTACAAGAAAGCATGCTCTGATTCTTCAAATACTGGATCTGTTGCCGAAGCTAATGCTCAGTTCTACCAGCAAGAATCCGCCAAACTCCGGAACCAAATCGAGGCTACGCAGAAGGGAATTAGGGACATGCTGGGTGAGAGTCTGAGCAGCTCAAGTGCCAGAGAACTCAAAAACCTAGAAGGTAAATTGGAGAAAGCAATCAGCAAAATCAGAACCAAAAAGAATGAGCTGTTGTTCCAAGAAATTGAGTACATGCAGAAAAGGCAAGTCGACTTGCACAACAATAACCAGCTTCTCCGAGCAAAGATAGCTGAAAATGAGAGAAGCCAGCAGAACATGAACGTGATGGCAGGAGGAGGAAGCTACGAGCTCATGCCGCAGTCTCAGGCGCAGACACAGGCATTCGACCCTTGGAATTATTTTCAAGTGAATGCCCTACAGCCGGCCAATCATCAGTACTCTCACCAAGATCCCATGGCCCTCCAATTAGTGTAA

## >MaAG1(XM_024164781.1-0)

AAACTCTTCTAATTACATTACTGATCATAATCATTTACTCTACTTCCTTTTGGCTTGTTATTGAGTATGGCGTACCAAAACAAGGCCATGATGTCAATGGATTCTCCGCAAAGGAAACTGGGAAGGGGGAAGATCGAGATTAAGAGGATCGAAAACACGACGAATCGTCAAGTAACCTTTTGCAAGCGGAGAAACGGCTTGTTGAAGAAGGCCTATGAACTCTCAGTTCTCTGTGATGCTGAGGTTGCTCTCATCGTCTTCTCTACCCGTGGACGTCTCTATGAGTATGCAAACCAGAGCGTTAAATCAACAATTGACAGGTACAAGAAAGCCTGCGCTGATTCTTCCAATACTGGATCTGTTGCCGAAATTAATGCTCAGTTCTACCAGAAAGAATCTGAAAAAATCCGTCAACAAATCGAGGCTACGCAGAAAGGAATCAGGGAAATGCTGGGTGATAGCGTGAGCAGTTCAAGTAACAGGGAACTCAAAAACCTGGAGGGTAAATTGGAGAAAGCAATTACAAAAATCAGAGCCAAAAAGAATGAGCTGCTGTTTCGAGAAATTGAGTACATGCAAAAAAGGGAAATCGACTTGCAATAGCTGAAAATGAGAGAGGCCAGCAGAACATGGACGTGATGGCAGGAGGTGGAAGCTACGAGCTCATGCCGCAGTCACAGCAGCAGCAGCAGAGTGTCTGA

## >MaAG2(XM_024174096.1-0)

ACGGAGTTCCCAAATCAAGCACCGGAAAGCTCTTCCCAGAGAAAAATGGGAAGAGGGAAAATTGAGATCAAGCGAATAGAGAACACAACCAACAGACAAGTCACTTTTTGTAAGCGCAGAAATGGTTTGCTCAAGAAAGCTTATGAGTTGTCTGTGTTGTGCGATGCTGAAGTTGCTCTTATTGTCTTCTCCAGCCGTGGACGCCTCTATGAGTACGCTAACAACAGCGTTAGAGGTACAATTGAAAGGTACAAGAAAGCATGTGCTGATTCTTCAAACACAGGATCTGTTGTCGAGGCCAATGTTCAGTTTTACCAGCAAGAAGCTTCCAAACTGAGAAGACAGATCCGGGAACTGCAGAACTCAAACAGGCATATACTGGGTGAAGCTCTAAGTTCTATGAGCTTAAAGGACCTCAAGAGCCTTGAAACAAGATTGGAGAAAGGAATCAGCAGAATCAGATCCAAGAAGAATGAAATGCTTTTTGCCGAAATCGAGATTATGCAGAAGAGGGAGATAGAGTTGCAAAGCCATAACAACTATCTGAGGGCAAAGATAAATGAACATGAGAGGATACAGCAGCAACAGGAGCAGCAGCAACACCAAATGAGCTTGATGCCGACCGGAACTGAATGCGAGTCCGTTCCTGCTTCGCAGTCGACTTATGATCGGAACTTTCTCCCTGTAAGCCTCATGAACACCAATCACCATTACTCTCGTCAAGACCAGACAGCTTTTCAACTTGTTTGAATTGCAGGAACCAGCAGGAAGGCGATTTGGTTTTATGGTTTTTATTATCTATATATATGCACTTCTAAATTGTAGCTTTATGTATAGACGATGTAAACAACTATTTTGTGCGTTTCCCACTATGTTGAAGGCGACTAATTTTCTCATGGCCAAACTCTTAAACTATTTGTTAGCTCAGCAGCATGCATCGAACTTTATTATGCACTCGTGTTATTAGAAAACATAAATAAGTACTTGATTAT

## >MaAG3(XM_010111053.2-0)

ATACCCACCCGCTAGACCAGGCCACTTCCTTTCCTATACATAACCAAAGAACTAAAAGAGACTCAACTACTCAAAGATCGATCAAGAAGAAGAACAAGTTGTTCAGCTTTCCTTTCTCCTCGGTTTTCCACTACAAATTTCTTTGGTTTTTTCTTTTCAAGACAGCAACCACGGAGTTCCCAAATCAAGCACCGGAAAGCTCTTCCCAGAGAAAAATGGGAAGAGGGAAAATTGAGATCAAGCGAATAGAGAACACAACCAACAGACAAGTCACTTTTTGTAAGCGCAGAAATGGTTTGCTCAAGAAAGCTTATGAGTTGTCTGTGTTGTGCGATGCTGAAGTTGCTCTTATTGTCTTCTCCAGCCGTGGACGCCTCTATGAGTACGCTAACAACAGCGTTAGAGGTACAATTGAAAGGTACAAGAAAGCATGTGCTGATTCTTCAAACACAGGATCTGTTGTCGAGGCCAATGTTCAGTTTTACCAGCAAGAAGCTTCCAAACTGAGAAGACAGATCCGGGAACTGCAGAACTCAAACAGGCATATACTGGGTGAAGCTCTAAGTTCTATGAGCTTAAAGGACCTCAAGAGCCTTGAAACAAGATTGGAGAAAGGAATCAGCAGAATCAGATCCAAGAAGAATGAAATGCTTTTTGCCGAAATCGAGATTATGCAGAAGAGGGAGATAGAGTTGCAAAGCCATAACAACTATCTGAGGGCAAAGATAAATGAACATGAGAGGATACAGCAGCAACAGGAGCAGCAGCAACACCAAATGAGCTTGATGCCGACCGGAACTGAATGCGAGTCCGTTCCTGCTTCGCAGTCGACTTATGATCGGAACTTTCTCCCTGTAAGCCTCATGAACACCAATCACCATTACTCTCGTCAAGACCAGACAGCTTTTCAACTTGTGTAA

# Protein sequence

## >FhRAN1(Fh.01G0000580)

MKRIQVGVAGMTCAACSNSVEAALMSVNGVLRASVALLQNKADVVFDPRLVKDEDIKNAIEDAGFEAEILPESSAVGTKPQATMLGQFSIGGMTCAACVNSVEGILRDLPGVKRAGVALATSLGEVEYDPAVISKEDIVNAIEDAGFEGAFVQSSEQDKIVLGVAGIYSEMDVQILGGILSSLKGVRQFHFDPISRELEVLFDPEVIHSRSLVDGVEGGSSGRFKLHVANPYSRMTSKDVEEASNMFRLFISSLFLSVPVFLIRVVCPHIPLIYSLLLWRCGPFQMGDWLKWALVSVVQFVVGKRFYIAAARALRNGSTNMDVLVALGTSASYFYSVCALLYGALTGFWSPTYFETSAMLITFVLLGKYLECLAKGKTSDAIKKLVELAPATATLLIKYKDGRYLGEREIDALLIQPGDTLKVSPGAKLPADGVVAWGSSYVNESMVTGESVPISKEVSSSVIGGTINLHGALHVQATKVGSDTVLSQIISLVETAQMSKAPIQKFADFIASIFVPTVVLLALLTLLGWYAAGALRAYPESWLPENGNYFVFALMFSISVVVIACPCALGLATPTAVMVATGVGANNGVLIKGGDALERAQKIKYVIFDKTGTLTQGKASVTTTKVFSGMDCGEFLKLVASAEASSEHPLAKAIVAYAHHFHFFDDSATKDAESHSKDSEVSGWLFDVAEFSALPGRGVQCFINEKQILVGNRKLMSESGIDIPDDVEKFVVELEESAKTGILVTYNGNLIGVLGVADPLKREAAVVVEGLSKMGIRPVMVTGDNWRTAQAVAKEVGIHDVRAEVMPAGKADVVRSFQKDGSTVAMVGDGINDSPALAAADVGMAIGAGTDIAIEAADYVLMRNNLEDVITAIDLSRKTFSRIRLNYVFAMAYNVIAIPVAAGVFFPSLGIQLPPWAAGACMALSSVSVVCSSLLLRRYRKPRLTTILEITVE

## >FmRAN1(Fm.01G0001630)

MLGQFSIGGMTCAACVNTVEGILRDLPGVKRAVVALATSLGEVEYDPAVISKEDIVNAIEDTGFEGAFLQSSEQDKIVLGVAGIYSEMDVQILGGILSNLKGVRQFHFDRISRELEVLFDPEVIHSRSLVDGVEGGSSGRFKLHVANPYSRMTSKDVEEASNMFRLFISSLFLSVPVFLIRVVCPHIPLIYSLLLWRCGPFQMGDWLKWALVSVVQFVVGKRFYIAAARALRNGSTNMDVLVALGTSASYFYSVCALLYGAFTGFWSPTYFETSAMLITFVLLGKYLECLAKGKTSDAIKKLVELAPATATLLIKDKDGRCLREREIDALLIQPGDTLKVSPGAKVPADGVVAWGSSYVNESMVTGESVPISKEVSSSVIGGTINLHGALHVQATKVGSDTVLSQIISLVETAQMSKAPIQKFADFIASIFVPTVVILALLTLMGWYAAGALGAYPESWLPENGNYFVFALMFSISVVVIACPCALGLATPTAVMVATGVGANNGVLIKGGDALERAQKIKYVIFDKTGTLTQGRASVTTTKVFSGMDRGEFLKLVASAEASSEHPLAKAIVAYARHFHFFDDSATKDAESHSKDSEVSGWLFDVTEFSTLPGRGVQCFINEKQILVGNRNLMTESGIDIPDDVEKFVVELEESAKTGILVSYNGNLIGVLGVADPLKREAAVVVEGLSKMGVRPVMVTGDNWRTAQAVAKEVGIHDVRAEVMPAGKADVVRSFQKDGSTVAMVGDGINDSPALAAADVGMAIGAGTDIAIEAADYVLMRNNLEDVITAIDLSRKTFFRIRLNYVFAMAYNVIAIPVAAGVFFPSLGIQLPPWAAGACMALSSVSVVCSSLLLRRYRKPRLTTILEITVE

## >AtRAN1-Q9S7J8

MAPSRRDLQLTPVTGGSSSQISDMEEVGLLDSYHNEANADDILTKIEEGRDVSGLRKIQVGVTGMTCAACSNSVEAALMNVNGVFKASVALLQNRADVVFDPNLVKEEDIKEAIEDAGFEAEILAEEQTQATLVGQFTIGGMTCAACVNSVEGILRDLPGVKRAVVALSTSLGEVEYDPNVINKDDIVNAIEDAGFEGSLVQSNQQDKLVLRVDGILNELDAQVLEGILTRLNGVRQFRLDRISGELEVVFDPEVVSSRSLVDGIEEDGFGKFKLRVMSPYERLSSKDTGEASNMFRRFISSLVLSIPLFFIQVICPHIALFDALLVWRCGPFMMGDWLKWALVSVIQFVIGKRFYVAAWRALRNGSTNMDVLVALGTSASYFYSVGALLYGAVTGFWSPTYFDASAMLITFVLLGKYLESLAKGKTSDAMKKLVQLTPATAILLTEGKGGKLVGEREIDALLIQPGDTLKVHPGAKIPADGVVVWGSSYVNESMVTGESVPVSKEVDSPVIGGTINMHGALHMKATKVGSDAVLSQIISLVETAQMSKAPIQKFADYVASIFVPVVITLALFTLVGWSIGGAVGAYPDEWLPENGTHFVFSLMFSISVVVIACPCALGLATPTAVMVATGVGATNGVLIKGGDALEKAHKVKYVIFDKTGTLTQGKATVTTTKVFSEMDRGEFLTLVASAEASSEHPLAKAIVAYARHFHFFDESTEDGETNNKDLQNSGWLLDTSDFSALPGKGIQCLVNEKMILVGNRKLMSENAINIPDHVEKFVEDLEESGKTGVIVAYNGKLVGVMGIADPLKREAALVVEGLLRMGVRPIMVTGDNWRTARAVAKEVGIEDVRAEVMPAGKADVIRSLQKDGSTVAMVGDGINDSPALAAADVGMAIGAGTDVAIEAADYVLMRNNLEDVITAIDLSRKTLTRIRLNYVFAMAYNVVSIPIAAGVFFPVLRVQLPPWAAGACMALSSVSVVCSSLLLRRYKKPRLTTVLKITTE

## >MnRAN1-W9SDE0

MAPNSRSLQLTQLSVSGAGDSGDLEEVRLLDAYENSEEEGVIGEGTMKRIQVGVTGMTCAACSNSVEAALMSVHGVLRASVALLQNKADVVFDPRLVKDEDIKSAIEDAGFEAEILPESSAVGTKPQGTLSGQFSIGGMTCAACVNSVEGILRDLPGVKRAVVALATSLGEVEYDPAIISKEDIVNAIEDAGFEGAFLQSSEQDKIVLGVAGIYSDVDVQLLGGILSNLKGMRQFYFDRITRELEVLFDPEVVNSRSLVDGIEGGSSGRFKLHVANPYSRMTSKDVEEASNMFRLFISSLFLSVPVFLIRVVCPHIPLIYSLLLWRCGPFQMGDWLKWALVSVVQFVVGKRFYIAAARALRNGSTNMDVLVALGTSASYFYSVCALLYGAVTGFWSPTYFETSAMLITFVLLGKYLECLAKGKTSDAIKKLVELAPATAMLLIKDKDGRCIGEREIDALLIQPGDTLKVLPGAKVPADGLVAWGTSYVNESMVTGESVPVSKQVGSRVIGGTINLHGALHIQATKVGSDTVLSQIISLVETAQMSKAPIQKFADFIASIFVPTVVMLALLTLLGWYMAGALGAYPESWLPENGNHFVFALMFSISVVVIACPCALGLATPTAVMVATGVGANNGVLIKGGDALERAQKIKYVIFDKTGTLTQGKASVTTTKVFTGMDRGEFLKLVASAEASSEHPLAKAIVAYAQHFHFFDDSAPKDAESNNKDSAVSGWLFDVAEFSALPGRGVQCFIDGKQILVGNRKLMTESGINIPDDVEKFVVDLEDSAKTGILVSYDGNLIGVLGVADPLKREAAVVVEGLSKMGVRPVMVTGDNWRTARAVAKEVGIHDVRAEVMPAGKADVIRSFQNDGSTVAMVGDGINDSPALAAADVGMAIGAGTDIAIEAADYVLMRSNLEDVITAIDLSRKTFSRIRLNYVFAMAYNVVAIPIAAGVFFPSSGIQLPPWAAGACMAMSSVSVVCSSLLLRRYRKPRLTTILEITVE

## >ZjRAN1-A0A6P4BE01

MAPSLKDLQLSQVVAAGRKSPAIVAGGDDSGDLEDVRLLDAYEASEGVEQGMKRIQVGVTGMTCAACSNSVEAALKSVNGVITASVALLQNKADVVFDPRLVKDEDIKNAIEDAGFEAEILSEPNATGTKPHGTLLGQFSIGGMTCAACVNSVEGILGNLPGVKKAVVALATSLGEVEYDPIMISKDDIVNAIEDAGFEASLVQSSEQDKILLGVTGISSEIDVQILESILSNLKGARQFYIDRNSRELEILFDPEVVNSRSLVYEIEGGSGGKFKLHVASPYTRMTSKDAEEASNMFRLFLSSLFLSIPVFLIRVVCPHIPLVYSLLLWQCGPFQMGDWLKWALVTLVQFGVGKRFYIAAGRALRNGSTNMDVLVALGTSASYFYSVCALLYGAFTGFWSPTYFETSAMLITFVLLGKYLECLAKGKTSDAIKKLVELAPATAMLIIKGKDGRCIGEREIDALLIQPGDTLKVLPGAKVPADGTVVWGSSYVNESMVTGESIPVLKEVGSSVIGGTINLHGALHIQATRVGSDAVLSQIISLVETAQMSKAPIQKFADFIASIFVPTVVSMALLTLLGWYAAGALGAYPADWLPVNGNHFVFALMFSISVVVIACPCALGLATPTAVMVATGVGANNGVLIKGGDALERAQKVKYVIFDKTGTLTQGKATVTSAKVFTGLDRGEFLKLVASAEVSSEHPLAKAIVEYARHFHFFDDSSATKDGEKHSKNSTIPEWLFDVSEFTSLPGRGVQCFIDGKRISVGNRKLMIECGIDIPTHVENYVVELEESAKTGILVAFNGKLIGVLGVADPLKREAAVVVEGLGKMGVRPVMVTGDNWRTARAVAKEVGIQDVQAEVMPAGKADVVRSFQRDGSVVAMVGDGINDSPALAAADVGMAIGAGTDIAIEAADYVLMRNNLEDVITAIDLSRKTFSRIRLNYVFAMAYNVIAIPVAAGLFYPSLGIKLPPWAAGACMALSSVSVVCSSLLLRRYKKPRLTTILEITVE

## >AtAG-P17839

MAYQSELGGDSSPLRKSGRGKIEIKRIENTTNRQVTFCKRRNGLLKKAYELSVLCDAEVALIVFSSRGRLYEYSNNSVKGTIERYKKAISDNSNTGSVAEINAQYYQQESAKLRQQIISIQNSNRQLMGETIGSMSPKELRNLEGRLERSITRIRSKKNELLFSEIDYMQKREVDLHNDNQILRAKIAENERNNPSISLMPGGSNYEQLMPPPQTQSQPFDSRNYFQVAALQPNNHHYSSAGRQDQTALQLV

## >BnAG1-Q01540

MAYQMELGGESSPQRKAGRGKIEIKRIENTTNRQVTFCKRRNGLLKKAYELSVLCDAEVALIVFSSRGRLYEYSNNSVKGTIERYKKAISDNSNTGSVAEINAQYYQQESAKLRQQIISIQNSNRQLMGETIGSMSPKELRNLEGRLDRSVNRIRSKKNELLFAEIDYMQKREVDLHNDNQLLRAKIAENERNNPSMSLMPGGSNYEQIMPPPQTQPQPFDSRNYFQVAALQPNNHHYSSAGREDQTALQLV

## >NtAG1-Q43585

MDFQSDLTREISPQRKLGRGKIEIKRIENTTNRQVTFCKRRNGLLKKAYELSVLCDAEVALIVFSSRGRLYEYANNSVKATIERYKKACSDSSNTGSISEANAQYYQQEASKLRAQIGNLQNQNRNMLGESLAALSLRDLKNLEQKIEKGISKIRSKKNELLFAEIEYMQKREIDLHNNNQYLRAKIAETERAQQQQQQQQMNLMPGSSSYELVPPPHQFDTRNYLQVNGLQTNNHYTRQDQPSLQLV

## >SlAG1-Q40168

MDFQSDLTREISPQRKLGRGKIEIKRIENTTNRQVTFCKRRNGLLKKAYELSVLCDAEVALVVFSNRGRLYEYANNSVKATIERYKKACSDSSNTGSVSEANAQYYQQEASKLRAQIGNLMNQNRNMMGEALAGMKLKELKNLEQRIEKGISKIRSKKNELLFAEIEYMQKREVDLHNNNQYLRAKIAETERAQHQHQQMNLMPGSSSNYHELVPPPQQFDTRNYLQVNGLQTNNHYPRQDQPPIQLV

## >VvMADS1-Q93XH4

MGRGKIEIKRIENTTNRQVTFCKRRNGLLKKAYELSVLCDAEVALIVFSSRGRLYEYANNSVRTTIERYKKVCSDSSNTGSVSEANAQFYQQEASKLRRQIRDIQNLNRHILGEALSSLNFKELKNLETRLEKGISRIRSKKNELLFAEIEYMQKREIELQNSNLFLRAQIAENERAQQQMNLMPGSQYESVPQQPYDSQNLLPVNLLDPNHHYSRHDQTALQLV

## >MnAG-W9SCS9

MYGQFYQQEASKLRRQIRELQNSNRHIVGEALSSMSLKDLKSLETRLEKGISRIRSKKNEMLFAEIEIMQKREIELQSHNNYLRAKINEHERIQQQQEQQQQQMSLMPTGTECESVPASQSTYDRNFLPVSLMNANHHYSRQDQTACRLV
