# Supplementary figures and images for: AGAMOUS Gene as a New Sex-Identification Marker in Fig (Ficus carica L.) Is More Efficient Than RAN1
Source: Front Plant Sci. 2021 Oct 20;12:755358. doi: 10.3389/fpls.2021.755358 (PMC8564383; doi:10.3389/fpls.2021.755358)

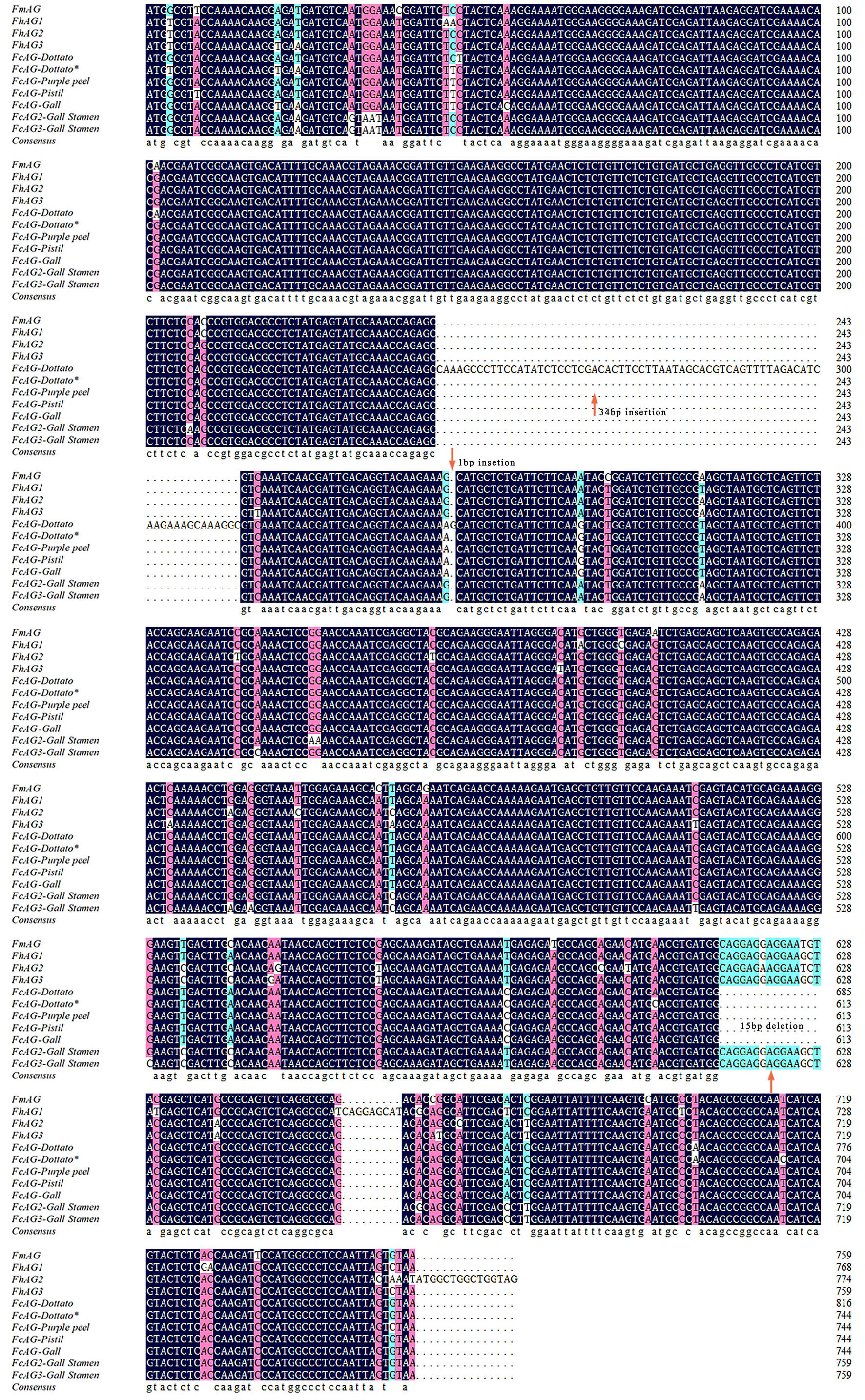

Supplement: Supplementary Figure 1 — Full-length alignment of Ficus AG genes. [file Data_Sheet_1.zip › Supplementary Figure 1.JPEG]

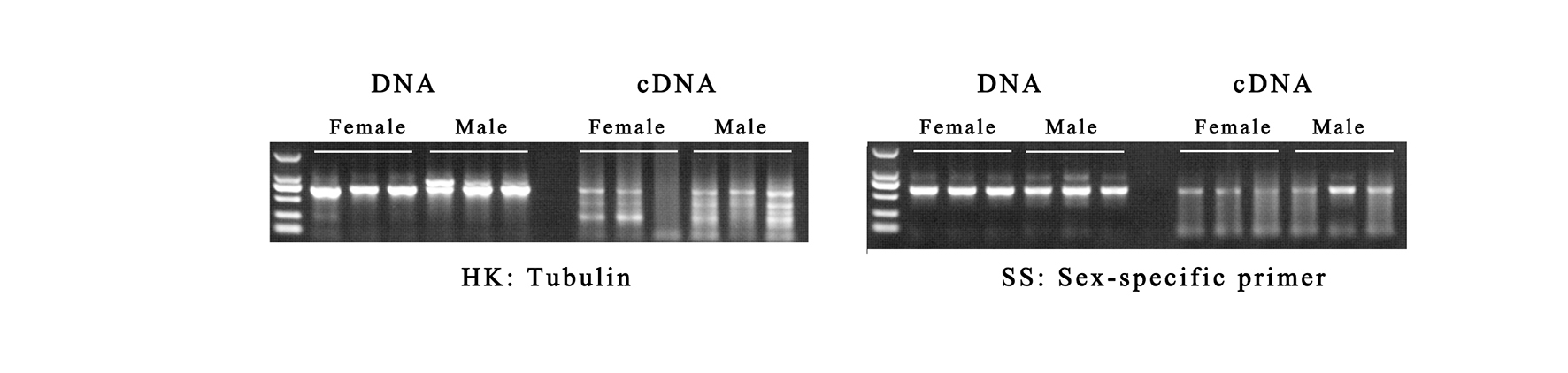

Supplement: Supplementary Figure 1 — Full-length alignment of Ficus AG genes. [file Data_Sheet_1.zip › Supplementary Figure 2.JPEG]

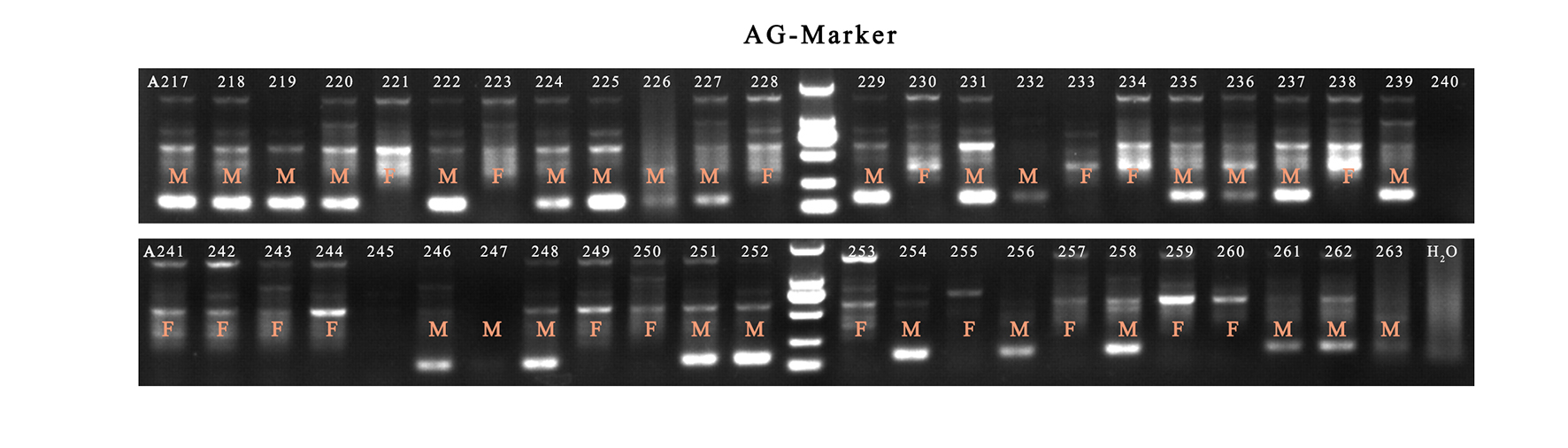

Supplement: Supplementary Figure 1 — Full-length alignment of Ficus AG genes. [file Data_Sheet_1.zip › Supplementary Figure 3.JPEG]

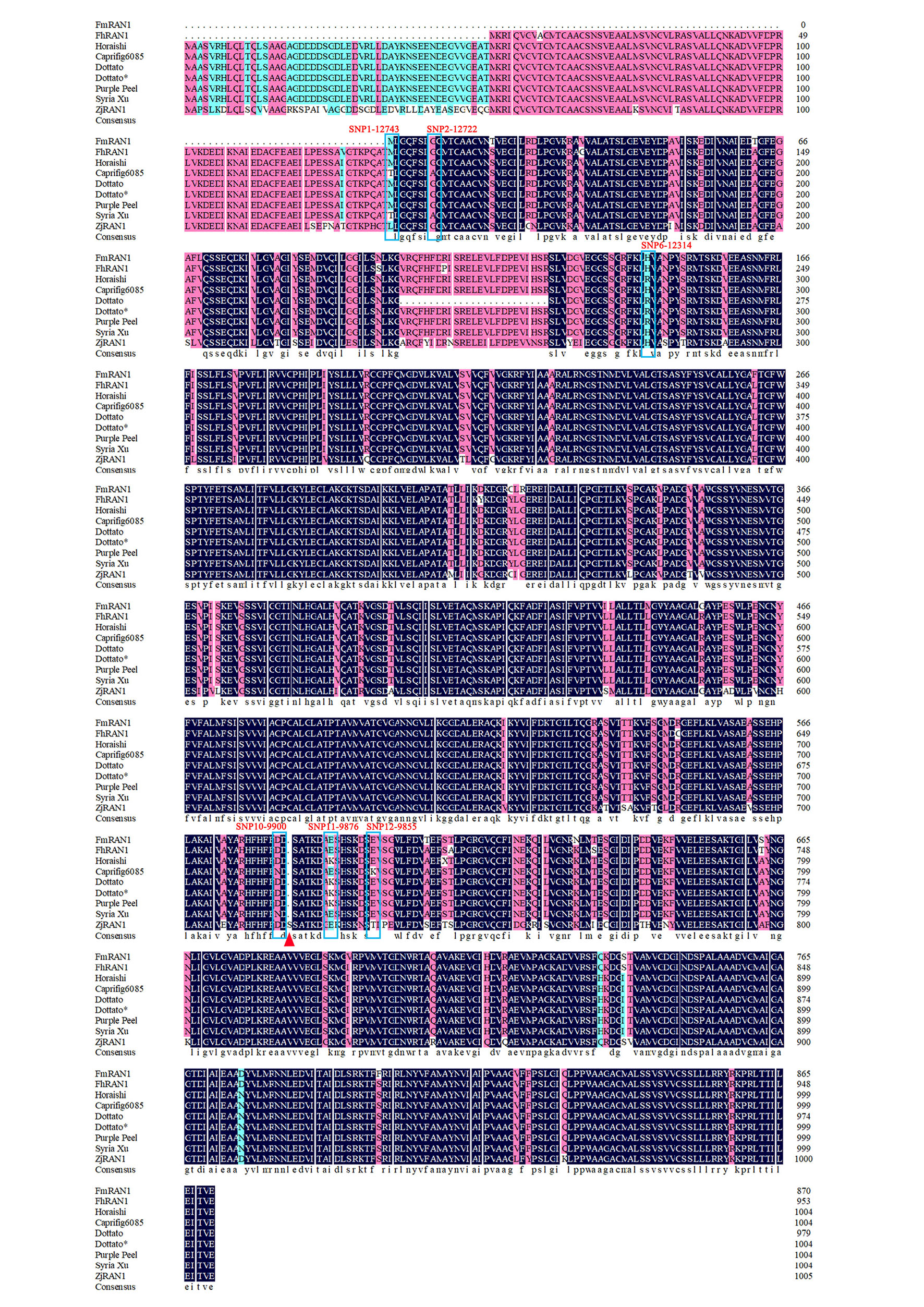

Supplement: Supplementary Figure 1 — Full-length alignment of Ficus AG genes. [file Data_Sheet_1.zip › Supplementary Figure 4.JPEG]
